# Adaptive Weighting-Based Multi-Station Doppler Orbit Determination for Noncooperative LEO Satellites

**DOI:** 10.3390/s26185932

**Published:** 2026-09-19

**Authors:** Ming Lei, Yue Liu, Zhihao Yang, Houhua Li, Zhibo Fang

**Affiliations:** 1 Aerospace Information Research Institute, Chinese Academy of Sciences, Beijing 100094, China; leiming20@mails.ucas.ac.cn (M.L.); liuyue212@mails.ucas.ac.cn (Y.L.); yangzhihao23@mails.ucas.ac.cn (Z.Y.); lihouhua24@mails.ucas.ac.cn (H.L.); 2 School of Electronic, Electrical and Communication Engineering, University of Chinese Academy of Sciences, Beijing 100049, China

**Keywords:** Iridium NEXT, Doppler orbit determination, multi-station observations, Levenberg–Marquardt damping, adaptive weighting

## Abstract

Precise orbit information is generally unavailable to users of noncooperative low Earth orbit (LEO) communication satellites such as Iridium NEXT, making publicly available two-line element (TLE) data the primary source of orbital states. However, TLE-derived orbital-state errors degrade Doppler positioning performance. This paper proposes an adaptively weighted multi-station Doppler orbit determination method. Using TLE-derived states as the orbit prior, the method formulates an orbital-dynamics-constrained batch least-squares (BLS) model. A priori weights are constructed from satellite elevation angles, and multi-station, multi-epoch Doppler measurements are adaptively reweighted by minimizing the uncertainty of the estimated satellite velocity projected onto the line of sight (LOS) from the ground station network centroid to the satellite. A Levenberg–Marquardt (LM)-type damping mechanism with diagonal scaling constrains the state correction vector to improve iterative stability. Experiments using real measurements show that the estimated orbital states yield a user-station two-dimensional root-mean-square error (2D RMSE) of 41.30 m, which is 16.06% lower than that obtained with the a priori weighted solution, 24.64% lower than that obtained with the equally weighted solution, and 64.25% lower than that obtained with the TLE-derived orbit. These results indicate that the proposed method improves the utility of the estimated orbital states for positioning with Iridium NEXT signals of opportunity.

## 1. Introduction

The Global Navigation Satellite System (GNSS) provides users worldwide with position, velocity, and timing information. It has been widely used in transportation, surveying and mapping, remote sensing, and emergency response. However, GNSS signals reach the Earth’s surface at low power and are vulnerable to multipath, blockage, and attenuation, degrading positioning performance in challenging environments such as urban canyons. To improve positioning in such environments, existing non-dedicated radio signals can be exploited. Navigation using these signals of opportunity (SOPs) has therefore attracted increasing attention [1]. Low Earth Orbit (LEO) satellite systems, including Starlink, OneWeb, Iridium, Globalstar, and ORBCOMM, have developed rapidly. Their communication downlink signals provide abundant resources for SOP-based navigation [2,3]. Compared with GNSS satellites, LEO satellites operate at lower altitudes, offer higher received signal power, and exhibit faster changes in satellite–ground geometry. These characteristics make LEO satellites promising for positioning applications in challenging environments [4,5,6].

Opportunistic positioning with LEO satellite signals typically estimates the user position using Doppler-shift, code-phase, or pseudorange measurements extracted from communication downlink signals. Some studies have also explored the use of carrier-phase measurements to further improve positioning or orbit estimation accuracy [7,8]. Previous studies have demonstrated the feasibility of using signals from LEO communication satellites, including Starlink, Iridium NEXT, and ORBCOMM, for opportunistic positioning [9,10,11,12,13,14]. However, unlike dedicated navigation satellites, LEO communication satellites are not primarily designed for navigation. Users without satellite-operator cooperation generally cannot obtain prior information such as precise ephemerides, satellite clock parameters, signal transmission times, and complete signal specifications. The resulting lack of complete orbit and time-frequency reference information is a major factor limiting opportunistic positioning performance with LEO satellite signals [15].

Satellite orbit errors are among the major error sources affecting Doppler positioning with LEO signals of opportunity. Doppler measurements reflect the relative velocity between the satellite and receiver along the line of sight (LOS). Their theoretical values depend on the satellite position and velocity, as well as the receiver state. When users without satellite-operator cooperation can only access publicly available two-line element (TLE) data, the satellite states must be propagated using the Simplified General Perturbations 4 (SGP4) model [16]. Orbit errors are then mapped into errors in the theoretical Doppler values and can subsequently introduce biases into the user position estimates. Wang et al. developed a model that maps LEO satellite orbit errors into equivalent Doppler measurement errors. Experiments using real Iridium NEXT and ORBCOMM signals demonstrated that orbit error compensation and weighting improved positioning accuracy [17]. Zhou et al. proposed a simultaneous positioning and orbit correction method to jointly estimate the user position and satellite orbit-error parameters [18]. Khairallah and Kassas analyzed the effects of LEO satellite ephemeris errors on opportunistic navigation from the perspective of error propagation. Their results indicated that ephemeris correction can improve subsequent positioning results [19].

Studies addressing the effects of orbit errors on positioning with LEO signals of opportunity can be broadly divided into two categories. The first compensates for or jointly estimates orbit errors during user positioning. The second uses external measurements to correct or predict the orbits of noncooperative LEO satellites. The first category mainly includes methods that compensate for errors during positioning, perform differential positioning, or jointly estimate the user position and orbit errors. Methods that compensate for orbit errors during positioning generally model them as equivalent measurement errors. Compensation or weighting strategies are then used to reduce their effects on the positioning results. However, these methods operate mainly within the positioning solution and cannot readily generate corrected orbital states that can be reused in subsequent periods [17]. Differential methods can use reference stations to mitigate some common errors. Saroufim et al. integrated differential LEO measurements with inertial navigation and improved navigation accuracy in the presence of TLE ephemeris errors [20,21]. However, in long-baseline positioning with LEO satellites, the LOS directions from the reference station and user station are not strictly parallel. Consequently, the spatial correlation of orbit and time-frequency errors in the differential measurements decreases. To address this issue, Wu et al. developed a geometric correction model for long-baseline differential Doppler positioning [22,23,24]. Simultaneous positioning and orbit correction methods can jointly estimate orbit errors during user positioning. However, they increase the number of parameters to be estimated, and their performance depends on the observation geometry, orbit-error parameterization, and prior constraints [18,25].

**Figure 1:**
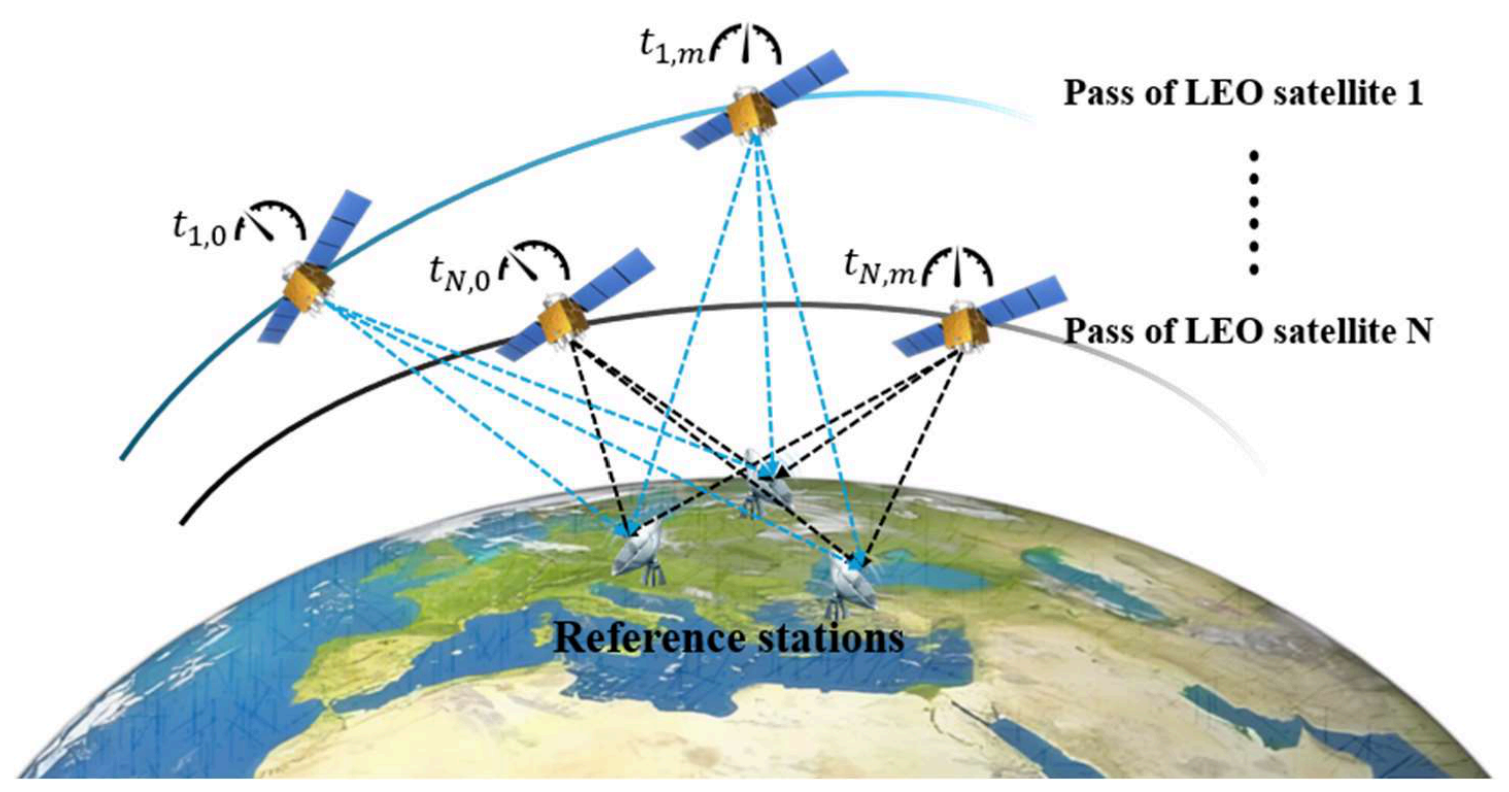
Schematic of LEO Satellite Orbit Determination Using Multi-Station Measurements of Signals of Opportunity.

In contrast to the methods described above, another category focuses on improving satellite orbital states rather than compensating for orbit errors during positioning. These studies use receiving stations at known locations for orbit estimation, orbit-error correction, or ephemeris prediction of noncooperative LEO satellites. The resulting orbital states are then used for subsequent positioning [26,27,28]. Deng et al. investigated orbit determination for noncooperative LEO satellites using single-pass Doppler measurements. Satellite orbits were estimated from Doppler shifts extracted from signals of opportunity [29]. Subsequently, Deng et al. proposed a single-station orbit determination framework for noncooperative LEO satellites in space-based opportunistic positioning. The framework performs initial orbit determination, precise orbit determination, and orbit prediction [30]. Haidar-Ahmad et al. initialized the satellite orbit using TLE data and employed a machine learning model to predict the ephemeris during out-of-view periods. The predicted ephemeris was then used for positioning during a subsequent pass [31]. Kassas et al. further proposed a closed-loop machine-learning orbit-prediction framework. They demonstrated that neural-network-predicted ephemerides improved opportunistic navigation results relative to SGP4-propagated ephemerides [32]. These studies indicate that publicly available orbital information can be used as a coarse prior and combined with real signal-of-opportunity measurements to generate enhanced orbital states. This provides a feasible approach to improving positioning with LEO signals of opportunity. Nevertheless, the performance of single-station Doppler orbit determination can still be affected by limited observation geometry and parameter correlations. Machine-learning-based ephemeris prediction, in contrast, depends on the training data and the model’s generalization capability [33]. Further research is therefore needed to fully exploit the spatial geometric constraints provided by multi-station Doppler measurements. This would enable stable estimation of noncooperative LEO satellite orbital states from TLE-derived initial orbits and support subsequent positioning with signals of opportunity. The scenario of LEO satellite orbit determination using multi-station signal-of-opportunity measurements is illustrated in Figure 1. Multiple ground reference stations at known locations receive LEO communication downlink signals either simultaneously or at different times. The extracted Doppler measurements are then used to constrain the satellite orbital states.

To this end, this paper proposes an orbit determination method for noncooperative LEO satellites using multi-station Doppler measurements. The proposed method uses orbital states derived from publicly available TLE data as the initial orbit prior. Multiple ground reference stations at known locations receive Iridium NEXT downlink signals, from which Doppler measurements are extracted. Under orbital dynamics constraints, a batch least-squares (BLS) framework is used to estimate the satellite orbital states and station clock-drift parameters. Weak constraints in some state-space directions can increase the condition number of the normal-equation matrix or even lead to ill-conditioning. Therefore, a Levenberg–Marquardt (LM)-type damping mechanism with diagonal scaling is introduced into the BLS state update to constrain the state correction vector and improve the stability of orbit determination iterations. However, LM damping primarily acts on the state update. It cannot distinguish either the quality of individual measurements or the strength of their geometric constraints on the orbital states. Conventional elevation- or signal-quality-based weighting mainly reflects differences in measurement quality and propagation conditions [34,35], whereas residual-based robust weighting primarily reduces the influence of measurements that are inconsistent with the current model [36,37,38]. Variance component estimation has also been widely used in satellite geodesy and combined orbit determination to determine relative weights among different stations or heterogeneous observation groups according to their stochastic characteristics [39,40,41,42]. Such approaches mainly adapt the weighting model based on the statistical characteristics of the observations or observation groups. More recently, refined stochastic modeling has also been used to explicitly account for physical correlations among observations in cooperative GNSS positioning, thereby improving measurement weighting and estimation reliability [43]. These approaches do not directly incorporate the contribution of individual Doppler measurements to the directional constraint on the estimated satellite velocity into the weighting criterion.

To address this limitation, the proposed method combines measurement-quality weighting with geometry-driven adaptive weighting. Elevation angles are first used to construct a priori weights that reflect measurement quality. The weights are then adaptively adjusted according to an information-based uncertainty metric for the estimated satellite velocity projected onto the LOS from the ground station network centroid to the satellite. In this way, the adaptive adjustment is driven by the contribution of each measurement to the Doppler-relevant directional constraint, rather than solely by elevation, signal quality, or residual magnitude.

Finally, the proposed method is validated through user-station positioning experiments using real Iridium NEXT signal-of-opportunity measurements.

## 2. BLS Orbit Determination Model Using Doppler Measurements

This section first considers a single reference station and develops a BLS orbit determination model using Doppler measurements. The model estimates the satellite orbital state at the initial epoch of the observation arc and the relative clock-drift parameter between the reference-station receiver and the satellite. Both parameters are iteratively updated using orbit propagation and the Doppler measurement model [29].

### 2.1. Parameterization and Orbital Dynamics

The Earth Mean Equator and Equinox of J2000.0 (EME2000) frame is adopted as the inertial reference frame. It provides a consistent representation of the satellite orbital state, third-body positions, and observation geometry. Accordingly, the satellite state vector is defined as:

(1) $$x\left(t\right)=\left[\begin{array}{c} P_{s}\left(t\right)\\ V_{s}\left(t\right) \end{array}\right]$$

where $P_{s}\left(t\right)={\left[\begin{array}{ccc} x_{s}\left(t\right) & y_{s}\left(t\right) & z_{s}\left(t\right) \end{array}\right]}^{T}$ and $V_{s}\left(t\right)={\left[\begin{array}{ccc} v_{s,x}\left(t\right) & v_{s,y}\left(t\right) & v_{s,z}\left(t\right) \end{array}\right]}^{T}$ denote the satellite position and velocity vectors, respectively.

The satellite equations of motion are expressed as

(2) $$\dot{x}\left(t\right)=f(x\left(t\right),t)=\left[\begin{array}{c} V_{s}\left(t\right)\\ a_{s}\left(t,P_{s}\left(t\right),V_{s}\left(t\right)\right) \end{array}\right]$$

where $a_{s}\left(t,P_{s}\left(t\right),V_{s}\left(t\right)\right)$ denotes the total acceleration acting on the satellite.

Given the state $x\left(t_{0}\right)={\left[\begin{array}{cc} {P_{s}}^{T}\left(t_{0}\right) & {V_{s}}^{T}\left(t_{0}\right) \end{array}\right]}^{T}$ at the initial epoch of the observation arc, the satellite orbit is numerically propagated using the orbital dynamics equation $\dot{x}\left(t\right)$. The satellite position and velocity at each observation epoch are then obtained. The state $x\left(t_{0}\right)$ at the initial epoch $t_{0}$ is treated as the unknown initial state in BLS orbit determination. It is iteratively estimated using the state transition matrix and the Doppler measurement model. The TLE-derived state is used only to initialize the orbit determination. During the BLS iterations, the six components of the initial satellite state are estimated and updated using the Doppler measurements, thereby partially correcting the initial orbital-state errors contained in the TLE-derived state [44].

### 2.2. State Transition Matrix Computation

In BLS orbit determination, the satellite states at different observation epochs are not estimated separately as independent unknowns. Instead, they are determined from a common initial state at the beginning of the observation arc, $t_{0}$. Therefore, a linear mapping is required between the state correction at each observation epoch and the initial-state correction at $t_{0}$. To establish this mapping, the state transition matrix (STM) $\Phi \left(t,t_{0}\right)$ is introduced to describe how changes in the initial state propagate to the state at time t. It is defined as:

(3) $$\Phi \left(t,t_{0}\right)=\frac{\partial x\left(t\right)}{\partial x\left(t_{0}\right)}$$

The STM satisfies the following variational equation:

(4) $$\begin{array}{l} \dot{\Phi }\left(t,t_{0}\right)=F\left(t\right)\Phi \left(t,t_{0}\right)\\ \Phi \left(t_{0},t_{0}\right)=I_{6} \end{array}$$

where $I_{6}$ is the 6×6 identity matrix, and $F\left(t\right)$ is the Jacobian matrix of the orbital dynamics equation with respect to the state vector, given by:

(5) $$F\left(t\right)=\frac{\partial f(x\left(t\right),t)}{\partial x\left(t\right)}=\left[\begin{array}{cc} 0_{3\times 3} & I_{3\times 3}\\ \frac{\partial a_{s}\left(t\right)}{\partial P_{s}\left(t\right)} & \frac{\partial a_{s}\left(t\right)}{\partial V_{s}\left(t\right)} \end{array}\right]$$

Accordingly, the state vector and the STM are combined into the following augmented system and integrated simultaneously:

(6) $$\left\{\begin{array}{l} \dot{x}\left(t\right)=f(x\left(t\right),t)\\ \dot{\Phi }\left(t,t_{0}\right)=F\left(t\right)\Phi \left(t,t_{0}\right) \end{array}\right.$$

Simultaneous integration of the state vector and the STM yields the satellite state at each observation epoch and its mapping to the state at $t_{0}$. These quantities are then combined with the reference station state to formulate the Doppler measurement equation.

### 2.3. Theoretical Doppler Measurement Model

For the *m*th Doppler measurement, let $t_{r,m}$ denote the signal reception epoch. Considering the finite signal propagation time, the corresponding transmission epoch $t_{t,m}$ is given by: $t_{t,m}=t_{r,m}-\tau_{m}$,$\tau_{m}=\frac{\left\|P_{r}\left(t_{r,m}\right)-P_{s}\left(t_{t,m}\right)\right\|}{c}$, where $\tau_{m}$ denotes the one-way signal propagation time. Because the satellite position depends on the transmission epoch, the propagation time is determined iteratively. An initial propagation time is first computed using the satellite state at the reception epoch. The satellite state at the corresponding transmission epoch is then updated, and the propagation time is recomputed until convergence [45].

The satellite position and velocity in the EME2000 frame at the transmission epoch are denoted by $P_{s}\left(t_{t,m}\right)={\left[\begin{array}{ccc} x_{s}\left(t_{t,m}\right) & y_{s}\left(t_{t,m}\right) & z_{s}\left(t_{t,m}\right) \end{array}\right]}^{T}$ and $V_{s}\left(t_{t,m}\right)={\left[\begin{array}{ccc} v_{s,x}\left(t_{t,m}\right) & v_{s,y}\left(t_{t,m}\right) & v_{s,z}\left(t_{t,m}\right) \end{array}\right]}^{T}$, respectively. The reference-station position and velocity in the EME2000 frame at the reception epoch are denoted by $P_{r}\left(t_{r,m}\right)={\left[\begin{array}{ccc} x_{r}\left(t_{r,m}\right) & y_{r}\left(t_{r,m}\right) & z_{r}\left(t_{r,m}\right) \end{array}\right]}^{T}$ and $V_{r}\left(t_{r,m}\right)={\left[\begin{array}{ccc} v_{r,x}\left(t_{r,m}\right) & v_{r,y}\left(t_{r,m}\right) & v_{r,z}\left(t_{r,m}\right) \end{array}\right]}^{T}$, respectively.

The Doppler measurement equation for the signal of opportunity is given by:

(7) $$f_{d}^{m}=\frac{f_{c}}{c}\cdot \frac{{\left(V_{s}\left(t_{t,m}\right)-V_{r}\left(t_{r,m}\right)\right)}^{T}\cdot \left(P_{r}\left(t_{r,m}\right)-P_{s}\left(t_{t,m}\right)\right)}{\left\|P_{r}\left(t_{r,m}\right)-P_{s}\left(t_{t,m}\right)\right\|}+f_{c}\delta \dot{t}+\varepsilon_{m}$$

where $f_{d}^{m}$ is the *m*th Doppler measurement, $f_{c}$ is the signal carrier frequency, and c is the speed of light. The term $\delta \dot{t}$ denotes the relative clock-drift parameter between the observation-station receiver and the satellite and represents their relative clock-rate error. It is dimensionless, while $f_{c}\delta \dot{t}$ represents the corresponding equivalent frequency bias in Hz. The term $\varepsilon_{m}$ represents the remaining error terms, including Doppler frequency measurement errors and unmodeled satellite-dependent frequency offsets. During each BLS iteration, the propagation time is recomputed using the updated orbital state, and the satellite position and velocity entering Equation (7) are updated accordingly.

### 2.4. BLS Estimation

Over the observation arc $\left[t_{0},t_{M}\right]$, the Doppler measurement equations are linearized using the theoretical Doppler measurement model. At each BLS iteration, the propagation time is first updated using the current orbital state. The converged transmission epoch is then used to evaluate the satellite state, STM, and measurement partial derivatives for the current linearization. The reference-station state is evaluated at the corresponding reception epoch. The STM maps the satellite-state corrections at the transmission epochs to $t_{0}$. BLS normal equations are then formed to jointly estimate the initial orbital state and clock-drift parameter, represented by $X\left(t_{0}\right)={\left[\begin{array}{cc} x^{T}\left(t_{0}\right) & \delta \dot{t} \end{array}\right]}^{T}$.

Let $h_{m}\left(X\left(t_{t,m}\right)\right)$ denote the theoretical Doppler measurement function, in which the satellite state is evaluated at the transmission epoch and the reference-station state is evaluated at the reception epoch. Then,

(8) $$f_{d}^{m}=h_{m}\left(X\left(t_{t,m}\right)\right)+\varepsilon_{m}$$

This gives:

(9) $$h_{m}\left(X\left(t_{t,m}\right)\right)=h_{m}\left(X^{\left(k\right)}\left(t_{t,m}\right)+\Delta X\left(t_{t,m}\right)\right)$$

where $X^{\left(k\right)}\left(t_{t,m}\right)=\left[x^{\left(k\right)}\left(t_{t,m}\right);\delta {\dot{t}}^{\left(k\right)}\right]$ is the parameter estimate at the kth iteration. The parameter correction between two consecutive iterations is $\Delta X\left(t_{t,m}\right)={\left[\begin{array}{cc} \Delta x^{T}\left(t_{t,m}\right) & \Delta \delta \dot{t} \end{array}\right]}^{T}$, with $\Delta x\left(t_{t,m}\right)=\left[\Delta P_{s}\left(t_{t,m}\right);\Delta V_{s}\left(t_{t,m}\right)\right]$.

Using the STM defined in Equation (3), the satellite state corrections at the transmission epoch $t_{t,m}$ and initial epoch $t_{0}$ satisfy:

(10) $$\Delta x\left(t_{t,m}\right)=\Phi \left(t_{t,m},t_{0}\right)\Delta x\left(t_{0}\right)$$

Because the observation arcs used in this study are short, with an average effective arc length of 11.43 min, the relative clock-drift parameter between the reference-station receiver and the satellite is approximated as constant within each arc. This parameter is jointly estimated with the orbital state to account for the relative clock-rate difference between the receiver and the satellite in the Doppler measurements. Thus, its estimate represents the receiver–satellite relative clock-rate error over the corresponding observation arc. Accordingly, the augmented parameter corrections at $t_{0}$ and $t_{t,m}$ satisfy:

(11) $$\Delta X\left(t_{m}\right)=\left[\begin{array}{cc} \Phi \left(t_{t,m},t_{0}\right) & 0_{6\times 1}\\ 0_{1\times 6} & 1 \end{array}\right]\Delta X\left(t_{0}\right)$$

where $\Delta X\left(t_{0}\right)={\left[\begin{array}{cc} \Delta x^{T}\left(t_{0}\right) & \Delta \delta \dot{t} \end{array}\right]}^{T}$ is the parameter correction vector to be estimated at $t_{0}$. The state correction at $t_{0}$ can be mapped to each transmission epoch using the STM. Thus, all unknown parameters over the observation arc can be expressed in terms of $\Delta X\left(t_{0}\right)$ and estimated jointly.

A first-order Taylor expansion of the measurement function $h_{m}\left(X\left(t_{t,m}\right)\right)$ for the *m*th Doppler measurement about the current estimate $X^{\left(k\right)}\left(t_{t,m}\right)$ gives:

(12) $$\begin{array}{l} h_{m}\left(X^{\left(k\right)}\left(t_{t,m}\right)+\Delta X\left(t_{t,m}\right)\right)\approx h_{m}\left(X^{\left(k\right)}\left(t_{t,m}\right)\right)+{\left.\frac{\partial h_{m}}{\partial \vec{X}}\right|}_{{\vec{X}}_{\left(t_{t,m}\right)}^{\left(k\right)}}\Delta X\left(t_{m}\right)\\ =h_{m}\left(X^{\left(k\right)}\left(t_{t,m}\right)\right)+{\left.\frac{\partial h_{m}}{\partial P_{s}\left(t_{t,m}\right)}\right|}_{X^{\left(k\right)}\left(t_{t,m}\right)}\Delta P_{s}\left(t_{t,m}\right)+\\ {\left.\frac{\partial h_{m}}{\partial V_{s}\left(t_{t,m}\right)}\right|}_{X^{\left(k\right)}\left(t_{t,m}\right)}\Delta V_{s}\left(t_{t,m}\right)+{\left.\frac{\partial h_{m}}{\partial \left(\delta \dot{t}\right)}\right|}_{X^{\left(k\right)}\left(t_{t,m}\right)}\Delta \left(\delta \dot{t}\right) \end{array}$$

Taking the partial derivatives with respect to the satellite position and velocity at the transmission epoch, together with the clock-drift parameter, gives:

(13) $$\begin{array}{c} \frac{\partial h_{m}}{\partial P_{s}\left(t_{t,m}\right)}=\frac{f_{c}}{cd_{m}}\cdot {\left(V_{s}\left(t_{t,m}\right)-V_{r}\left(t_{r,m}\right)\right)}^{T}\left(u_{m}u_{m}^{T}-I_{3}\right)\\ \frac{\partial h_{m}}{\partial V_{s}\left(t_{t,m}\right)}=\frac{f_{c}}{c}u_{m}^{T}\;\;\;\;\;\;\;\;\;\;\;\;\;\;\;\;\;\;\;\;\;\;\;\;\;\;\;\;\;\;\;\;\;\;\;\;\;\;\;\;\;\;\;\;\;\;\;\;\;\;\;\;\;\;\;\;\;\;\;\;\;\;\\ \frac{\partial h_{m}}{\partial \left(\delta \dot{t}\right)}=f_{c}\;\;\;\;\;\;\;\;\;\;\;\;\;\;\;\;\;\;\;\;\;\;\;\;\;\;\;\;\;\;\;\;\;\;\;\;\;\;\;\;\;\;\;\;\;\;\;\;\;\;\;\;\;\;\;\;\;\;\;\;\;\;\;\; \end{array}$$

where $d_{m}=\left\|P_{r}\left(t_{r,m}\right)-P_{s}\left(t_{t,m}\right)\right\|$ and $u_{m}=\frac{P_{r}\left(t_{r,m}\right)-P_{s}\left(t_{t,m}\right)}{d_{m}}$.

Therefore, the linearized measurement equation for the *m*th Doppler measurement is expressed as:

(14) $$G_{m}\Delta X\left(t_{0}\right)=b_{m}$$

where

(15) $$\begin{array}{l} G_{m}=\left[\begin{array}{cc} \left[\begin{array}{cc} \frac{\partial h_{m}}{\partial P_{s}\left(t_{t,m}\right)} & \frac{\partial h_{m}}{\partial V_{s}\left(t_{t,m}\right)} \end{array}\right]\Phi \left(t_{t,m},t_{0}\right) & f_{c} \end{array}\right]\\ b_{m}=f_{d}^{m}-h_{m}\left(X^{\left(k\right)}\left(t_{t,m}\right)\right)\;\;\;\;\;\;\;\;\;\;\;\;\;\;\;\;\;\;\;\;\;\;\;\;\;\;\;\;\;\;\;\;\;\;\;\;\;\;\;\;\;\;\;\;\;\;\;\;\;\;\;\;\;\;\;\;\;\;\;\;\;\;\;\;\;\;\;\;\;\;\;\;\;\;\;\;\;\;\;\;\;\;\;\;\;\;\;\;\;\;\;\;\;\;\;\;\;\;\;\; \end{array}$$

Stacking the linearized equations for all M measurements over the observation arc yields the complete linearized measurement system:

(16) $$G\Delta X\left(t_{0}\right)=b$$

where

(17) $$G=\left[\begin{array}{c} G_{1}\\ G_{2}\\ \vdots \\ G_{M} \end{array}\right]\;b=\left[\begin{array}{c} b_{1}\\ b_{2}\\ \vdots \\ b_{M} \end{array}\right]$$

The least-squares solution for the state correction is:

(18) $$\Delta X\left(t_{0}\right)={\left(G^{T}G\right)}^{-1}G^{T}b$$

The parameter estimate from the *k*th iteration is then updated as follows:

(19) $$X^{\left(k+1\right)}\left(t_{0}\right)=X^{\left(k\right)}\left(t_{0}\right)+\Delta X^{\left(k\right)}\left(t_{0}\right)$$

After each iteration, the orbit is numerically propagated again from the updated initial state, while the STM is integrated simultaneously. The ground station position and velocity in the inertial frame are also recomputed. This process continues until convergence, yielding the satellite orbital state and clock-drift parameter at the initial epoch of the observation arc.

## 3. Orbit Determination Workflow and Model Configuration

This section describes the implementation of LEO satellite orbit determination using Doppler measurements. The overall workflow first covers orbit initialization, state propagation, measurement equation formulation, and BLS iteration. The reference frames and coordinate transformations, orbital dynamics model configuration, and numerical orbit propagation method are then described.

### 3.1. Orbit Determination Workflow

**Figure 2:**
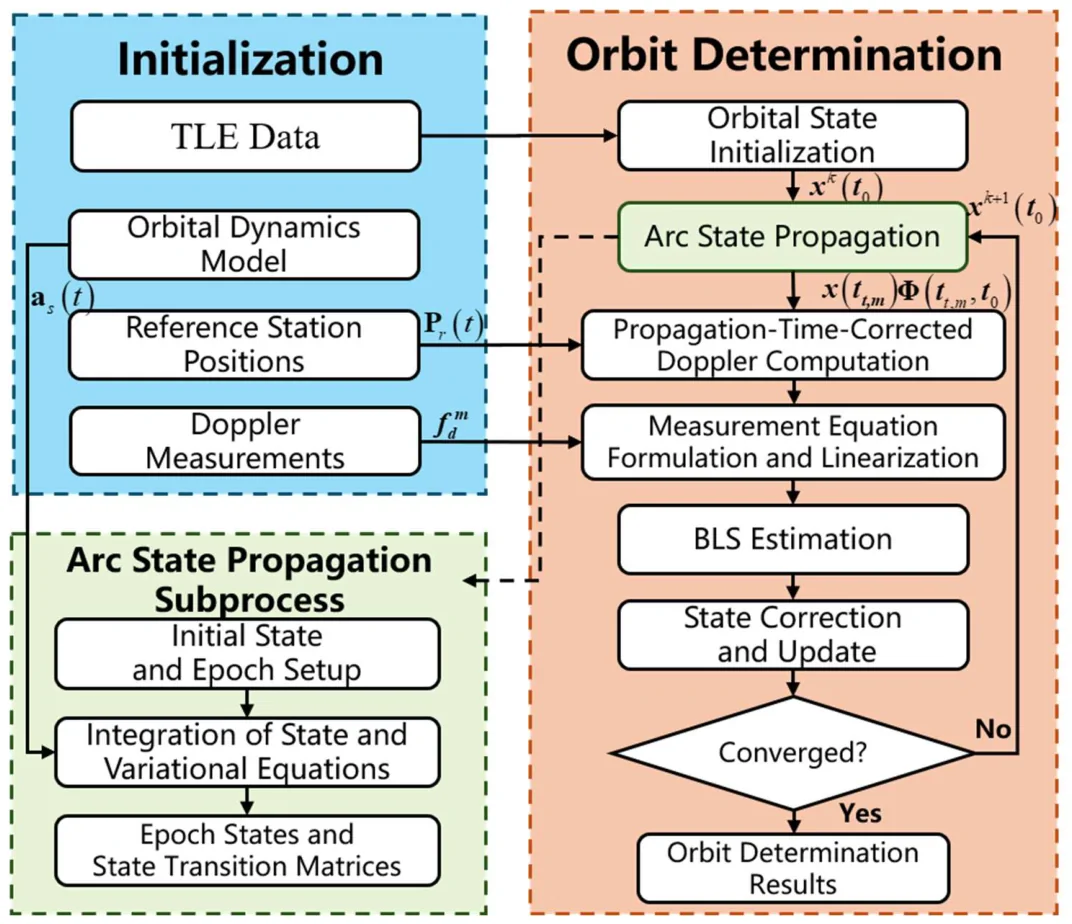
Flowchart of LEO Satellite Orbit Determination Using Doppler Measurements.

The overall orbit determination workflow is shown in Figure 2. First, Doppler measurements are extracted from the Iridium NEXT downlink signals received by the reference station. The initial orbital state at the beginning of the arc is obtained from publicly available Celestrak TLE data using SGP4 and transformed from the TEME frame to EME2000 before orbit determination. Next, the orbital dynamics model is configured, and the reference station coordinates are transformed into the inertial frame to obtain its position and velocity at each reception epoch. For each Doppler measurement, the propagation time is updated using the current orbital state, and the satellite state and STM are evaluated at the corresponding converged transmission epoch. The propagation-time-corrected satellite state, the reference-station state, and the measured Doppler data are then used to formulate and linearize the Doppler measurement equations. BLS estimation is applied to estimate the initial orbital state and clock-drift parameter. If convergence is not achieved, state propagation, measurement equation formulation, and parameter estimation are repeated using the updated orbital state. The orbit determination results are output after convergence. The following subsections describe the reference frames and coordinate transformations, orbital dynamics model configuration, and state propagation method.

### 3.2. Reference Frames and Coordinate Transformations

The satellite position and velocity obtained from TLE data using SGP4 are initially expressed in the True Equator, Mean Equinox (TEME) frame and are transformed into the EME2000 frame as

(20) $$P_{s}^{\mathrm{EME}2000}\left(t\right)=C_{T\to E}\left(t\right)P_{s}^{\mathrm{TEME}}\left(t\right)$$

(21) $$V_{s}^{\mathrm{EME}2000}\left(t\right)=C_{T\to E}\left(t\right)V_{s}^{\mathrm{TEME}}\left(t\right)+{\dot{C}}_{T\to E}\left(t\right)P_{s}^{\mathrm{TEME}}\left(t\right)$$

where $C_{T\to E}\left(t\right)$ denotes the TEME-to-EME2000 rotation matrix. The TLE epoch is expressed in UTC, and the TEME-to-EME2000 transformation follows the IAU-76/FK5 formulation.

The reference station coordinates are initially given in the International Terrestrial Reference Frame (ITRF), whereas the satellite state is propagated in the EME2000 inertial frame. Therefore, the station coordinates are transformed into the same inertial frame according to the IERS Conventions published by the International Earth Rotation and Reference Systems Service (IERS) [46].

Let $P_{g}^{\mathrm{ITRF}}\left(t\right)$ denote the reference station position vector in the ITRF. Its position in the EME2000 frame is expressed as:

(22) $$P_{g}^{\mathrm{EME}2000}\left(t\right)=BQ\left(t\right)R\left(t\right)W\left(t\right)P_{g}^{\mathrm{ITRF}}\left(t\right)$$

where $W\left(t\right)$, $R\left(t\right)$, and $Q\left(t\right)$ are the rotation matrices for polar motion, Earth rotation, and precession–nutation, respectively. The Earth-rotation and polar-motion components are determined using IERS Earth orientation parameters, including polar motion, the difference between UT1 and UTC, and the length of day. The matrix B is the constant bias rotation matrix from the Geocentric Celestial Reference System to the EME2000 frame. The time derivative of Equation (22) gives the reference station velocity in the EME2000 frame.

The ground reference station is assumed fixed in the ITRF. Its inertial-frame velocity is therefore governed mainly by frame rotations associated with Earth rotation, polar motion, and precession–nutation. The resulting position and velocity in the EME2000 frame at each observation epoch are used for subsequent orbital dynamics and Doppler measurement modeling.

### 3.3. Orbital Dynamics Model Configuration

The orbital dynamics model underpins LEO satellite orbit propagation and parameter estimation. The propagation model accounts for the Earth’s gravity field, third-body perturbations from the Sun and Moon, atmospheric drag, tidal perturbations, solar radiation pressure, and relativistic corrections. The total satellite acceleration is therefore expressed as:

(23) $$a_{s}\left(t\right)=a_{\mathrm{grav}}\left(t\right)+a_{3b}\left(t\right)+a_{\mathrm{drag}}\left(t\right)+a_{\mathrm{tide}}\left(t\right)+a_{\mathrm{srp}}\left(t\right)+a_{\mathrm{rel}}\left(t\right)$$

where $a_{\mathrm{grav}}\left(t\right)$, $a_{3b}\left(t\right)$, $a_{\mathrm{drag}}\left(t\right)$, $a_{\mathrm{tide}}\left(t\right)$, $a_{\mathrm{srp}}\left(t\right)$, and $a_{\mathrm{rel}}\left(t\right)$ denote the accelerations due to the Earth’s gravity field, third-body perturbations from the Sun and Moon, atmospheric drag, tidal perturbations, solar radiation pressure, and relativistic corrections, respectively.

#### 3.3.1. Earth Gravity Field Model

The Earth’s gravity field is the dominant force in satellite orbit propagation. The EIGEN-6S Earth gravity field model is used and truncated at degree and order 60. Its gravitational potential is expressed as:

(24) $$U_{\mathrm{grav}}\left(t\right)=\frac{\mu }{r}\left[1+\sum_{n=2}^{60}{\left(\frac{R_{e}}{r}\right)}^{n}\sum_{m=0}^{n}{\bar{P}}_{nm}\left(\sin\varphi \right)\left({\bar{C}}_{nm}\left(t\right)\cos m\lambda +{\bar{S}}_{nm}\left(t\right)\sin m\lambda \right)\right]$$

where μ is the Earth’s gravitational parameter, and $R_{e}$ is the reference Earth radius. The variables r, φ, and λ denote the geocentric distance, geocentric latitude, and longitude, respectively. The term ${\bar{P}}_{nm}\left(\sin\varphi \right)$ is the normalized associated Legendre function, while ${\bar{C}}_{nm}\left(t\right)$ and ${\bar{S}}_{nm}\left(t\right)$ are the normalized spherical harmonic coefficients. The indices n and m denote the degree and order, respectively, with 0≤m≤n.

The acceleration due to the Earth’s gravity is obtained by taking the negative gradient of the potential with respect to the satellite position vector ${\vec{P}}_{s}\left(t\right)$:

(25) $$a_{\mathrm{grav}}\left(t\right)=-\nabla U_{\mathrm{grav}}\left(t\right)$$

#### 3.3.2. Third-Body Perturbation Model for the Sun and Moon

In addition to the Earth’s gravity, orbit propagation accounts for third-body perturbations from the Sun and Moon. These perturbations are modeled using the differential gravitational formulation as:

(26) $$a_{3b}\left(t\right)=\sum_{b=1}^{n_{p}}\mu_{b}\left(\frac{P_{b}\left(t\right)-P_{s}\left(t\right)}{{\left\|P_{b}\left(t\right)-P_{s}\left(t\right)\right\|}^{3}}-\frac{P_{b}\left(t\right)}{{\left\|P_{b}\left(t\right)\right\|}^{3}}\right)$$

where $P_{b}\left(t\right)$ denotes the position vector from the geocentric position vector to the *b*th third body. It is obtained from the DE440 planetary and lunar ephemeris released by the Jet Propulsion Laboratory. The variable $n_{p}$ denotes the number of third bodies included in the calculation. In this study, $n_{p}=2$, corresponding to the Sun and Moon. The parameter $\mu_{b}$ is the gravitational parameter of the *b*th third body [47].

#### 3.3.3. Atmospheric Drag Model

Atmospheric drag is an important nonconservative perturbation in LEO satellite orbit propagation. The atmospheric drag acceleration is modeled using the conventional aerodynamic formulation:

(27) $$a_{\mathrm{drag}}\left(t\right)=-\frac{1}{2}C_{d}\frac{A}{m}\rho \left(P_{s}\left(t\right),t\right)\left\|V_{rel}\left(t\right)\right\|V_{rel}\left(t\right)$$

where $C_{d}$ is the drag coefficient, and A/m is the cross-sectional-area-to-mass ratio. The atmospheric density $\rho \left(P_{s}\left(t\right),t\right)$ is computed using the NRLMSISE-00 model, and $V_{rel}\left(t\right)$ is the satellite velocity vector relative to the atmosphere [48].

#### 3.3.4. Tidal Perturbation Model

Tidal perturbations are included in orbit propagation to account for temporal variations in the Earth’s gravity field. Both solid Earth tides and ocean tides are considered. Solid Earth tides are modeled following the IERS Conventions (2010), while ocean tides are modeled using the FES2004 global ocean tide model. Both effects are incorporated through time-dependent corrections to the spherical harmonic coefficients of the Earth’s gravity field. The additional gravitational potential due to tidal effects is expressed as:

(28) $$\Delta U_{\mathrm{tide}}\left(t\right)=\frac{\mu }{r}\sum_{n=2}^{12}{\left(\frac{R_{e}}{r}\right)}^{n}\sum_{m=0}^{n}{\bar{P}}_{nm}\left(\sin\varphi \right)\left[\Delta {\bar{C}}_{nm}^{\mathrm{tide}}\left(t\right)\cos m\lambda +\Delta {\bar{S}}_{nm}^{\mathrm{tide}}\left(t\right)\sin m\lambda \right]$$

where $\Delta {\bar{C}}_{nm}\left(t\right)$ and $\Delta {\bar{S}}_{nm}\left(t\right)$ are the tide-induced corrections to the normalized spherical harmonic coefficients. These corrections include contributions from solid Earth tides and ocean tides. The tidal perturbation acceleration is obtained by taking the negative gradient of the additional potential with respect to the satellite position vector ${\vec{P}}_{s}\left(t\right)$:

(29) $$a_{\mathrm{tide}}\left(t\right)=-\nabla \Delta U_{\mathrm{tide}}\left(t\right)$$

#### 3.3.5. Solar Radiation Pressure Model

The structural and optical properties of noncooperative LEO satellites are generally not accurately known. Therefore, the solar radiation pressure acceleration is modeled using the classical cannonball model:

(30) $$a_{\mathrm{srp}}\left(t\right)=\delta P_{0}C_{R}\frac{A}{m}{\left(\frac{\mathrm{AU}}{\left\|P_{s}\left(t\right)-r_{sun}\left(t\right)\right\|}\right)}^{2}\frac{P_{s}\left(t\right)-r_{sun}\left(t\right)}{\left\|P_{s}\left(t\right)-r_{sun}\left(t\right)\right\|}$$

where δ is the shadow function indicating whether the satellite is within the Earth’s shadow. AU denotes the astronomical unit, representing the mean Earth–Sun distance. The parameter $P_{0}$ is the solar radiation pressure constant at 1 AU, and $C_{R}$ is the reflectivity coefficient. The ratio A/m is the satellite’s illuminated-area-to-mass ratio, and $r_{sun}\left(t\right)$ is the position vector of the Sun in the inertial frame.

#### 3.3.6. Relativistic Correction Model

Relativistic corrections are modeled using the Schwarzschild model based on the first-order post-Newtonian approximation:

(31) $$a_{\mathrm{rel}}\left(t\right)=\frac{\mu }{c^{2}{\left\|P_{s}\left(t\right)\right\|}^{3}}\left[\left(4\frac{\mu }{\left\|P_{s}\left(t\right)\right\|}-{V_{s}}^{T}\left(t\right)V_{s}\left(t\right)\right)P_{s}\left(t\right)+4\left({P_{s}}^{T}\left(t\right)V_{s}\left(t\right)\right)V_{s}\left(t\right)\right]$$

This model accounts for the dominant general relativistic correction to orbital motion in the Earth’s central gravitational field. Other relativistic terms are not modeled because of their smaller magnitudes.

### 3.4. Numerical Orbit Propagation

The satellite state is propagated using a Dormand–Prince 8(5,3) explicit Runge–Kutta integrator. This yields the satellite position and velocity at each observation epoch within the observation arc [49].

Let $t_{m}$ and $t_{m+1}$ denote two consecutive observation epochs. Integration over $\left[t_{m},t_{m+1}\right]$ is performed using multiple steps, where n indexes the integration steps within this interval. For the *n*th integration step, $x\left(t_{n}\right)$ denotes the state vector at $t_{n}$. The stage quantities for this step are defined as:

(32) $$k_{i}=f\left(t_{n}+c_{i}h,x\left(t_{n}\right)+h\sum_{j=1}^{i-1}a_{ij}k_{j}\right),\;i=1,\ldots ,s$$

where h is the integration step size, s is the number of stages, and $k_{i}$ is the slope vector at the *i*th stage. The parameters $a_{ij}$ and $c_{i}$ are the Butcher coefficients of the Dormand–Prince 8(5,3) method.

The state is updated using the eighth-order weights, yielding the state vector $x^{\left(8\right)}\left(t_{n+1}\right)$ at $t_{n+1}$:

(33) $$x^{\left(8\right)}\left(t_{n+1}\right)=x\left(t_{n}\right)+h\sum_{i=1}^{s}b_{i}^{\left(8\right)}k_{i}$$

where $b_{i}^{\left(8\right)}$ is the weight coefficient for the eighth-order solution. Multiple integration steps are performed between consecutive observation epochs. The satellite state is thereby propagated from the initial epoch of the arc to each observation epoch, yielding the state vectors throughout the arc.

## 4. Multi-Station Doppler Orbit Determination with Adaptive Weighting

The preceding BLS model is extended to joint multi-station orbit determination. An adaptive-weighting method for multi-station Doppler orbit determination is then proposed. Satellite elevation angles are used to construct a priori measurement weights. Directional uncertainty is characterized using an information-based metric for the estimated satellite velocity projected onto the LOS from the station network centroid to the satellite. The multi-station, multi-epoch Doppler measurement weights are then adjusted adaptively. Unlike the elevation-based a priori weights, which characterize measurement quality, the adaptive adjustment factors are updated using the sensitivity of the velocity-projection uncertainty metric with respect to each measurement’s adjustment factor, thereby incorporating the contribution of each Doppler measurement to the Doppler-relevant directional constraint on the estimated satellite velocity into the weighting process. An LM-type damping mechanism with diagonal scaling is also introduced to stabilize the BLS state update. This mechanism limits potentially excessive state corrections along weakly constrained directions.

**Figure 3:**
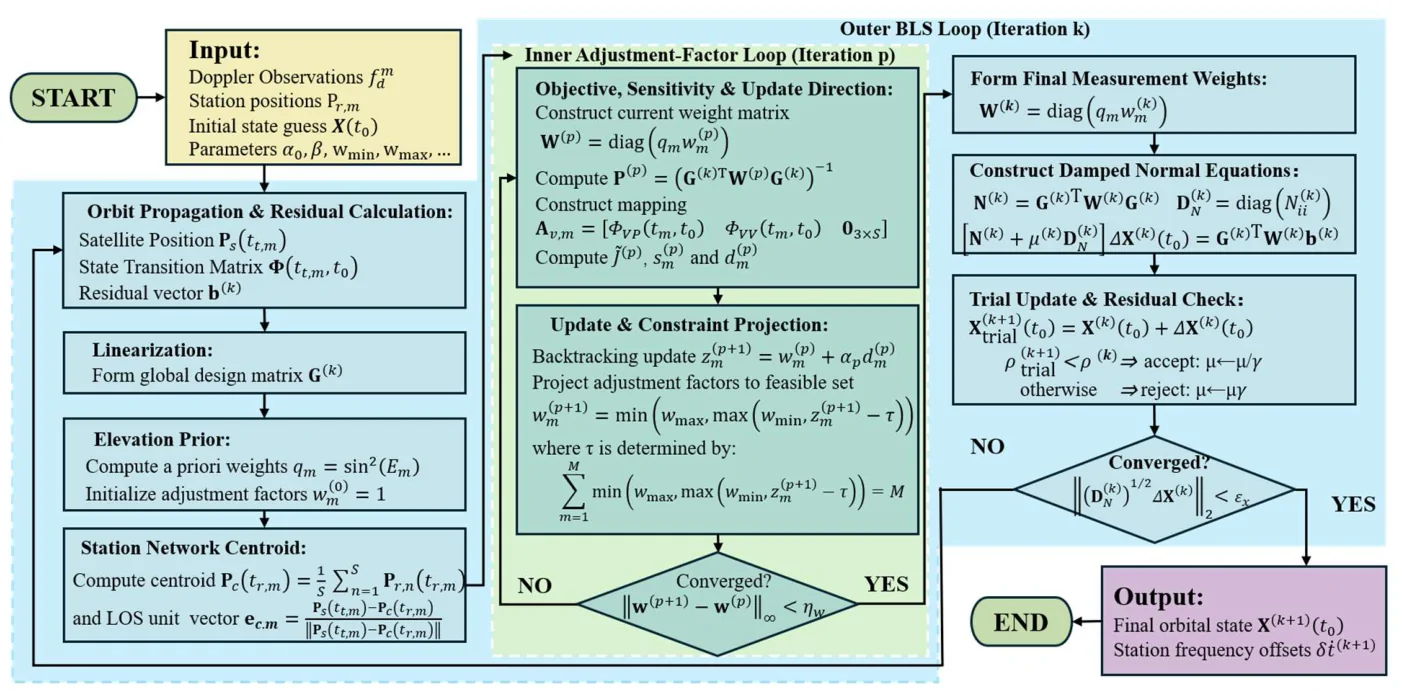
Flowchart of Multi-Station Doppler Orbit Determination with Adaptive Weighting.

Figure 3 shows the overall workflow of the proposed method. First, the elevation-based a priori weights are constructed, and the LOS direction from the station network centroid to the satellite is computed. The regularized adjustment-factor optimization objective is then evaluated. The adaptive adjustment factors are updated using the projected-gradient method with backtracking line search. The final measurement weights are obtained by multiplying the elevation-based a priori weights by the optimized adaptive adjustment factors. The resulting weight matrix is used in the weighted BLS state update with an LM-type damping mechanism with diagonal scaling.

### 4.1. Construction of Elevation-Based a Priori Weights

In multi-station Doppler orbit determination for LEO satellites, observation geometry and propagation conditions vary with elevation angle. These differences affect the stability and reliability of Doppler measurements. Low-elevation measurements are more susceptible to blockage, multipath effects, and variations in propagation conditions. They are therefore assigned lower a priori weights. A sine-function model is used to construct the elevation-based a priori weight for the *m*th measurement:

(34) $$q_{m}=\sin^{2}\left(E_{m}\right)$$

where $E_{m}$ is the satellite elevation angle associated with the *m*th Doppler measurement, and $q_{m}$ is its elevation-based a priori weight. A higher elevation angle gives a larger $q_{m}$ and thus a higher a priori weight in orbit determination.

The final measurement weight matrix is defined as:

(35) $$W=\mathrm{diag}\left(q_{m}w_{m}\right)$$

where $w_{m}$ is the adaptive adjustment factor to be optimized. The final measurement weight $q_{m}w_{m}$ is the product of the elevation-based a priori weight $q_{m}$ and the adaptive adjustment factor $w_{m}$. The adjustment factor adjusts the relative contribution of the measurement according to the velocity-projection objective. The adjustment factors are initialized as $w_{m}^{\left(0\right)}=1$, m=1,2,…M, where M is the total number of measurements. Thus, before adaptive adjustment, the final measurement weights are equal to the elevation-based a priori weights.

### 4.2. Construction of the LOS Direction from the Network Centroid

Since the orbital state is expressed in the inertial frame, the Earth-fixed coordinates of each station are transformed into inertial coordinates at the corresponding reception epoch. For the *m*th Doppler measurement, let $P_{r,n}\left(t_{r,m}\right)$ denote the inertial-frame position of station *n* at the reception epoch.

The network centroid is defined as:

(36) $$P_{c}\left(t_{r,m}\right)=\frac{1}{S}\sum_{n=1}^{S}P_{r,n}\left(t_{r,m}\right)$$

where S is the number of stations involved in orbit determination.

The LOS unit vector from the network centroid to the satellite is:

(37) $$e_{c,m}=\frac{P_{s}\left(t_{t,m}\right)-P_{c}\left(t_{r,m}\right)}{\left\|P_{s}\left(t_{t,m}\right)-P_{c}\left(t_{r,m}\right)\right\|}$$

where $P_{s}\left(t_{t,m}\right)$ is the inertial-frame position of the satellite at the corresponding transmission epoch. To further characterize the geometric representativeness of the centroid LOS, let $\Delta P_{n,m}=P_{r,n}\left(t_{r,m}\right)-P_{c}\left(t_{r,m}\right)$ denote the position offset of station n from the network centroid, and let $\rho_{c,m}=\left\|P_{s}\left(t_{t,m}\right)-P_{c}\left(t_{r,m}\right)\right\|$ denote the satellite slant range from the network centroid. For a regional station network whose characteristic spatial extent L is small relative to $\rho_{c,m}$, the LOS direction from each station can be expanded about the network centroid as

(38) $$\begin{array}{c} e_{n,m}=e_{c,m}-\frac{\left(I-e_{c,m}e_{c,m}^{T}\right)\Delta P_{n,m}}{\rho_{c,m}}+O\left(\frac{L^{2}}{\rho_{c,m}^{2}}\right)\\ \frac{1}{S}\sum_{n=1}^{S}e_{n,m}=e_{c,m}+O\left(\frac{L^{2}}{\rho_{c,m}^{2}}\right) \end{array}$$

where $e_{n,m}$ denotes the LOS unit vector from station n to the satellite, and $L=\max_{n}\left\|\Delta P_{n,m}\right\|$ characterizes the spatial extent of the station network. By the definition of the network centroid, $\sum_{n=1}^{S}\Delta P_{n,m}=0$; therefore, the first-order station-offset terms cancel when averaged over the network. For a common velocity-uncertainty matrix at a given epoch, the same cancellation applies to the first-order variation in the corresponding velocity-projection uncertainty. Accordingly, the velocity-projection uncertainty along the centroid LOS provides a first-order approximation to the average velocity-projection uncertainty along the individual station LOS directions. Therefore, the centroid LOS is adopted as a common representative direction for constructing the network-level weight-optimization metric.

### 4.3. Formulation of the Velocity-Projection Uncertainty Metric

Measurements provide different geometric constraints on the satellite orbital state. To relate satellite orbital-state errors to Doppler measurements, a first-order perturbation of the satellite position and velocity is considered. The corresponding orbit-induced Doppler error for the *m*th measurement can be approximated as

(39) $$\delta f_{d,m}^{orb}\approx H_{P,m}\delta P_{s}\left(t_{t,m}\right)+H_{V,m}\delta V_{s}\left(t_{t,m}\right)$$

where

(40) $$H_{P,m}=\frac{\partial h_{m}}{\partial P_{s}\left(t_{t,m}\right)},H_{V,m}=\frac{\partial h_{m}}{\partial V_{s}\left(t_{t,m}\right)}$$

If the orbital-state error covariance at the transmission epoch is denoted by

(41) $$\sum_{orb,m}=\left[\begin{array}{cc} \sum_{PP,m} & \sum_{PV,m}\\ \sum_{VP,m} & \sum_{VV,m} \end{array}\right]$$

the corresponding first-order Doppler-error variance can be expressed as

(42) $$\begin{array}{cc} \sigma_{f,m,orb}^{2} & =H_{P,m}\sum_{PP,m}H_{P,m}^{T}+H_{V,m}\sum_{VV,m}H_{V,m}^{T}\\ & +H_{P,m}\sum_{PV,m}H_{V,m}^{T}+H_{V,m}\sum_{VP,m}H_{P,m}^{T} \end{array}$$

This formulation includes the effects of satellite position errors, velocity errors, and their cross-covariance. According to Equation (13), the direct first-order contribution of the satellite velocity error is

(43) $$H_{V,m}\delta V_{s}\left(t_{t,m}\right)=\frac{f_{c}}{c}u_{m}^{T}\delta V_{s}\left(t_{t,m}\right)$$

which is proportional to the velocity-error component projected onto the LOS direction. Based on this Doppler sensitivity characteristic, the proposed weighting scheme uses an information-based uncertainty metric for the estimated satellite velocity projected onto the LOS from the station network centroid to the satellite as the weight-optimization metric. Satellite position information is simultaneously incorporated into the BLS estimation through the position partial derivatives in the design matrix and the position–velocity coupling represented by the STM.

Based on the weighted BLS model, an information-based uncertainty matrix for the augmented state at the reference epoch is defined as:

(44) $$P_{0}\left(W\right)={\left[G^{T}\mathrm{diag}\left(q_{m}w_{m}\right)G\right]}^{-1}$$

where G is the design matrix assembled from all linearized measurement equations. This matrix is used to characterize the relative uncertainty of the estimated state under different measurement-weight configurations. In joint multi-station orbit determination, the clock-drift parameter of each station is treated as an independent unknown. The augmented state vector at the reference epoch is therefore extended as:

(45) $$X\left(t_{0}\right)=\left[x^{T}(t_{0}),\delta {\dot{t}}_{0},\ldots ,\delta {\dot{t}}_{S}\right]\in {\mathbb{R}}^{6+S}$$

where S is the number of stations involved in orbit determination. Therefore, the design matrix G has dimensions $M\times \left(6+S\right)$.

Let $a\in {\mathbb{R}}^{S}$ denote the station-selection vector for the *m*th Doppler measurement. The element corresponding to the ground station from which the measurement is obtained is one, and all other elements are zero. The *m*th row of the multi-station design matrix is therefore written as:

(46) $$G_{m}=\left[\begin{array}{cc} \left[\begin{array}{cc} \frac{\partial h_{m}}{\partial P_{s}\left(t_{t,m}\right)} & \frac{\partial h_{m}}{\partial V_{s}\left(t_{t,m}\right)} \end{array}\right]\Phi \left(t_{t,m},t_{0}\right) & f_{c}a_{m}^{T} \end{array}\right]$$

Thus, each Doppler measurement is associated only with the clock-drift parameter of the corresponding station. The station clock-drift parameters are not propagated through the orbital dynamics. Accordingly, the mapping matrix from the augmented state at the reference epoch to the satellite velocity at each transmission epoch is constructed as follows:

(47) $$A_{v,m}=\left[\begin{array}{ccc} \Phi_{VP}\left(t_{t,m},t_{0}\right) & \Phi_{VV}\left(t_{t,m},t_{0}\right) & 0_{3\times S} \end{array}\right]$$

where $\Phi_{VP}\left(t_{t,m},t_{0}\right)$ maps the initial position estimation error to the velocity estimation error at the transmission epoch. Similarly, $\Phi_{VV}\left(t_{t,m},t_{0}\right)$ maps the initial velocity estimation error to the velocity estimation error at that epoch. The zero matrix $0_{3\times S}$ indicates that the station clock-drift parameters have no dynamical effect on the propagated satellite velocity.

The corresponding information-based uncertainty matrix for the satellite velocity at transmission epoch $t_{t,m}$ is given by:

(48) $$P_{v}\left(t_{t,m}\right)=A_{v,m}P_{0}\left(W\right)A_{v,m}^{T}$$

The velocity-projection uncertainty metric along the LOS from the network centroid to the satellite is then formulated as:

(49) $$J\left(W\right)=\sum_{m=1}^{M}e_{c,m}^{T}A_{v,m}P_{0}\left(W\right)A_{v,m}^{T}e_{c,m}$$

This metric characterizes the relative uncertainty of the estimated satellite velocity projected onto the representative LOS direction of the station network over the entire observation arc. A smaller $J\left(W\right)$ indicates lower relative uncertainty in the estimated velocity projected along this direction.

Here, $J\left(W\right)$ is an information-based relative uncertainty metric. According to Equation (43), the direct first-order sensitivity of a Doppler measurement to satellite velocity is governed by the velocity component projected onto the corresponding station-to-satellite LOS. As shown in Section 4.2, the centroid LOS provides a first-order representative direction for the individual station LOS directions in a regional station network. Therefore, $J\left(W\right)$ provides a first-order surrogate for the Doppler-relevant velocity component of orbital-state uncertainty.

The downstream effect of the corresponding orbit-induced Doppler errors on user-state estimation can then be described through local first-order error propagation. Let $\delta z_{D}^{orb}$ denote the Doppler-error vector induced by satellite orbital-state errors. The corresponding perturbation in the estimated user state and its covariance contribution can be expressed as

(50) $$\delta {\hat{x}}_{u}^{orb}=K_{u}\delta z_{D}^{orb},{\sum_{u}^{orb}=K}_{u}R_{D}^{orb}K_{u}^{T}$$

where $R_{D}^{orb}$ is the covariance matrix of the orbit-induced Doppler errors, and $K_{u}$ denotes the Jacobian that locally maps perturbations in the Doppler observations to perturbations in the estimated user state. Therefore, reducing the uncertainty of the Doppler-relevant velocity component reduces a first-order surrogate of the orbit-induced Doppler uncertainty and is expected to reduce the corresponding contribution to the user-state uncertainty. Accordingly, the proposed velocity-projection uncertainty metric provides a first-order indicator of the influence of orbit-induced Doppler uncertainty on user-state estimation. The final positioning performance is additionally affected by satellite position errors, user–satellite geometry, measurement noise, and other error sources.

To construct the regularized objective used in the adjustment-factor update, the normalized velocity-projection uncertainty metric is combined with a quadratic regularization term:

(51) $$\tilde{J}\left(w\right)=\frac{J\left(W\right)}{J_{\mathrm{ref}}}+\frac{\beta }{2M}\sum_{m=1}^{M}{\left(w_{m}-1\right)}^{2}$$

where $w={\left[w_{1},w_{2},\ldots w_{M}\right]}^{T}$ denotes the adaptive adjustment-factor vector, $J_{\mathrm{ref}}$ is the value of $J\left(W\right)$ at the beginning of the current inner adjustment-factor optimization loop, and β is the regularization coefficient.

### 4.4. Computation of the Weight-Optimization Gradient

After formulating the velocity-projection uncertainty metric, the effect of each adaptive adjustment factor on the objective is evaluated. The analytical gradient of the objective function with respect to the adaptive adjustment factors quantifies the sensitivity of the objective to each factor.

To prevent a uniform increase in all adjustment factors from artificially reducing the uncertainty metric, the adaptive adjustment factors $w_{m}$ are subject to a sum constraint and upper and lower bounds:

(52) $$C_{w}=\left\{\begin{array}{cc} w:\sum_{m=1}^{M}w_{m}=M, & w_{\min}\leq w_{m}\leq w_{\max} \end{array}\right\}$$

where $w_{\min}$ and $w_{\max}$ are the lower and upper bounds of the adaptive adjustment factors, respectively. These constraints redistribute the adaptive adjustment factors among the measurements. They also prevent the objective function from being artificially reduced by uniformly increasing all adjustment factors.

The analytical gradient of the velocity-projection uncertainty metric with respect to the adaptive adjustment factor $w_{m}$ of the *m*th measurement is given by:

(53) $$g_{m}=\frac{\partial J\left(W\right)}{\partial w_{m}}=-q_{m}{\sum_{j=1}^{M}\left[G_{m}P_{0}\left(W\right)A_{v,j}^{T}e_{c,j}\right]}^{2}$$

where $G_{m}$ is the row vector of the design matrix G corresponding to the *m*th measurement. The index j denotes the *j*th Doppler measurement whose contribution to the velocity-projection uncertainty metric is accumulated in the objective function.

The sensitivity of the velocity-projection uncertainty metric to the *m*th measurement is defined as:

(54) $$s_{m}=-g_{m}=q_{m}{\sum_{j=1}^{M}\left[G_{m}P_{0}\left(W\right)A_{v,j}^{T}e_{c,j}\right]}^{2}$$

At the current iteration, a larger $s_{m}$ indicates that increasing the adaptive adjustment factor of the *m*th measurement produces a larger local decrease in the velocity-projection uncertainty metric along the LOS from the network centroid to the satellite. The provisional update depends on both this sensitivity and the regularization term, while the final adjustment factor is further affected by the constraint projection.

### 4.5. Adjustment-Factor Update Using the Projected Gradient Method

After this sensitivity is obtained, the adaptive adjustment factors are updated along the descent direction of the regularized objective. The provisional adjustment factors may violate the sum and bound constraints. Projection and clipping are therefore applied to restore feasibility.

During the *k*th BLS iteration, the current design matrix $G^{\left(k\right)}$, velocity mapping matrix $A_{v,m}$, and LOS direction $e_{c,m}$ from the network centroid are held fixed. The adaptive adjustment factors are updated through a series of inner iterations. Let $w_{m}^{\left(p\right)}$ denote the adaptive adjustment factor of the *m*th measurement at the *p*th inner iteration of the adjustment-factor update process. At each inner iteration, the final measurement weight matrix is first constructed as:

(55) $$W^{\left(p\right)}=\mathrm{diag}\left(q_{m}w_{m}^{\left(p\right)}\right)$$

The current information-based uncertainty matrix is then computed as:

(56) $$P_{0}^{\left(p\right)}={\left(G^{\left(k\right)T}W^{\left(p\right)}G^{\left(k\right)}\right)}^{-1}$$

Using the current information-based uncertainty matrix, $s_{m}^{\left(p\right)}$ is evaluated according to Equation (54). The update direction of the regularized objective is then given by:

(57) $$d_{m}^{\left(p\right)}=\frac{s_{m}^{\left(p\right)}}{J_{ref}}-\frac{\beta }{M}\left(w_{m}^{\left(p\right)}-1\right)$$

The provisional adjustment factor is then updated as:

(58) $$z_{m}^{\left(p+1\right)}=w_{m}^{\left(p\right)}+\alpha_{p}d_{m}^{\left(p\right)}$$

where $\alpha_{p}$ is the step size obtained through backtracking line search with $\alpha_{0}$ as the initial value.

Projection and clipping are then applied to the provisional adjustment factor:

(59) $$w_{m}^{\left(p+1\right)}=\min\left(w_{\max},\max\left(w_{\min},z_{m}^{\left(p+1\right)}-\tau \right)\right)$$

where τ is determined by the sum constraint:

(60) $$\sum_{m=1}^{M}\min\left(w_{\max},\max\left(w_{\min},z_{m}^{\left(p+1\right)}-\tau \right)\right)=M$$

The value of τ is obtained using a one-dimensional bisection search. When the change in the adjustment factors satisfies:

(61) $${\left\|w^{\left(p+1\right)}-w^{\left(p\right)}\right\|}_{\infty }<\eta_{w}$$

The adjustment-factor update within the current BLS iteration is then terminated. This yields the adaptive adjustment factor vector $w^{\left(k\right)}$ for the *k*th BLS iteration. Here, $\eta_{w}$ is the convergence threshold for the adjustment-factor iterations. The optimized adjustment factors are multiplied by the corresponding elevation-based a priori weights to form the final measurement weights used in the subsequent weighted BLS state update with diagonally scaled LM-type damping.

### 4.6. State Update with Diagonally Scaled Damping

The final measurement weight matrix for the *k*th iteration is formed by multiplying each adaptive adjustment factor by the corresponding elevation-based a priori weight:

(62) $$W^{\left(k\right)}=\mathrm{diag}\left(q_{1}w_{1}^{\left(k\right)},q_{2}w_{2}^{\left(k\right)},\cdots ,q_{M}w_{M}^{\left(k\right)}\right)$$

In Doppler orbit determination, the estimation sensitivity of each state component depends on the observation-arc length, station distribution, satellite–ground geometry, and measurement weights. LM-type damping with diagonal scaling is used to suppress excessive corrections along weakly constrained directions and stabilize the weighted BLS state update [50].

Let the weighted normal matrix at the *k*th iteration be:

(63) $$N^{\left(k\right)}=G^{\left(k\right)T}W^{\left(k\right)}G^{\left(k\right)}$$

The diagonal scaling matrix is constructed from its diagonal elements as:

(64) $$D_{N}^{\left(k\right)}=\mathrm{diag}\left(N_{11}^{\left(k\right)},N_{22}^{\left(k\right)},\cdots ,N_{n_{x}n_{x}}^{\left(k\right)}\right)$$

where $n_{x}$ is the dimension of the augmented state vector. The weighted normal equations with diagonally scaled damping are then written as [50]:

(65) $$\left[N^{\left(k\right)}+\mu^{\left(k\right)}D_{N}^{\left(k\right)}\right]\Delta X^{\left(k\right)}\left(t_{0}\right)=G^{\left(k\right)T}W^{\left(k\right)}b^{\left(k\right)}$$

where $\mu^{\left(k\right)}$ is the LM damping factor at the *k*th iteration. The damping term is constructed from the diagonal elements of the normal matrix, thereby accounting for the numerical scale differences among state components.

After obtaining the state correction, the trial state is constructed as:

(66) $$X_{\mathrm{trial}}^{\left(k+1\right)}\left(t_{0}\right)=X^{\left(k\right)}\left(t_{0}\right)+\Delta X^{\left(k\right)}\left(t_{0}\right)$$

To adaptively adjust the damping factor, the weighted residual sums of squares for the current and trial states are defined as:

(67) $$\begin{array}{c} \rho^{\left(k\right)}=r^{\left(k\right)T}W^{\left(k\right)}r^{\left(k\right)}\\ \rho_{\mathrm{trial}}^{\left(k+1\right)}=r_{\mathrm{trial}}^{\left(k+1\right)T}W^{\left(k\right)}r_{\mathrm{trial}}^{\left(k+1\right)} \end{array}$$

where $r^{\left(k\right)}$ and $r_{\mathrm{trial}}^{\left(k+1\right)}$ are the measurement residual vectors for the current and trial states, respectively. The corresponding weighted residual sums of squares are denoted by $\rho^{\left(k\right)}$ and $\rho_{\mathrm{trial}}^{\left(k+1\right)}$. During the evaluation of the trial state, the weight matrix is fixed at $W^{\left(k\right)}$, as determined for the current iteration. This prevents coupling between the damping-factor adjustment and the weight update.

Based on the change in the weighted residual sum of squares for the trial state, the state and damping factor are updated as follows:

(68) $$\left\{\begin{array}{l} X^{\left(k+1\right)}\left(t_{0}\right)=X_{\mathrm{trial}}^{\left(k+1\right)}\left(t_{0}\right),\mu^{\left(k+1\right)}=\frac{\mu^{\left(k\right)}}{\gamma },\rho_{\mathrm{trial}}^{\left(k+1\right)}<\rho^{\left(k\right)}\\ X^{\left(k+1\right)}\left(t_{0}\right)=X^{\left(k\right)}\left(t_{0}\right),\mu^{\left(k+1\right)}=\gamma \mu^{\left(k\right)},\rho_{\mathrm{trial}}^{\left(k+1\right)}\geq \rho^{\left(k\right)} \end{array}\right.$$

where γ>1. If the trial state reduces the weighted residual sum of squares, it is accepted and the damping factor is decreased; otherwise, the current state is retained and the damping factor is increased for the next iteration. Convergence is not assessed when the trial state is rejected.

State convergence is assessed only after the trial state is accepted. The diagonally scaled state correction is used as the convergence criterion:

(69) $${\left\|{\left(D_{N}^{\left(k\right)}\right)}^{1/2}\Delta X^{\left(k\right)}\left(t_{0}\right)\right\|}_{2}<\varepsilon_{x}$$

where $\varepsilon_{x}$ is the convergence threshold for the diagonally scaled state correction. If convergence is achieved, the iteration stops and returns the satellite orbital state and station clock-drift parameters at the initial epoch of the arc. Otherwise, the accepted state becomes the new linearization point, and orbit propagation, state transition matrix integration, residual computation, weight update, and damped state-correction computation are repeated.

In summary, the proposed method uses the information-based uncertainty of the satellite velocity projected along the LOS from the station network centroid as the weight-optimization metric. Based on this metric, the adjustment-factor optimization objective is constructed by combining the normalized velocity-projection uncertainty metric with a quadratic regularization term. The final measurement weights are obtained by multiplying the elevation-based a priori weights by the optimized adaptive adjustment factors. An LM-type damping mechanism with diagonal scaling constrains the state corrections during the weighted batch least-squares update.

## 5. Experimental Results and Analysis

This section experimentally evaluates the proposed multi-station Doppler orbit determination method using real Iridium NEXT signals of opportunity. The experimental setup and the iterative behavior of diagonally scaled LM are first presented. The characteristics of the measured multi-station Doppler observations, adaptive weight allocation, and the effect of the number of reference stations are then analyzed. Finally, the estimated orbital states are evaluated using in-arc post-fit Doppler residuals, user-station positioning results, and subsequent-pass user-station Doppler residuals.

### 5.1. Experimental Setup and Parameters

**Figure 4:**
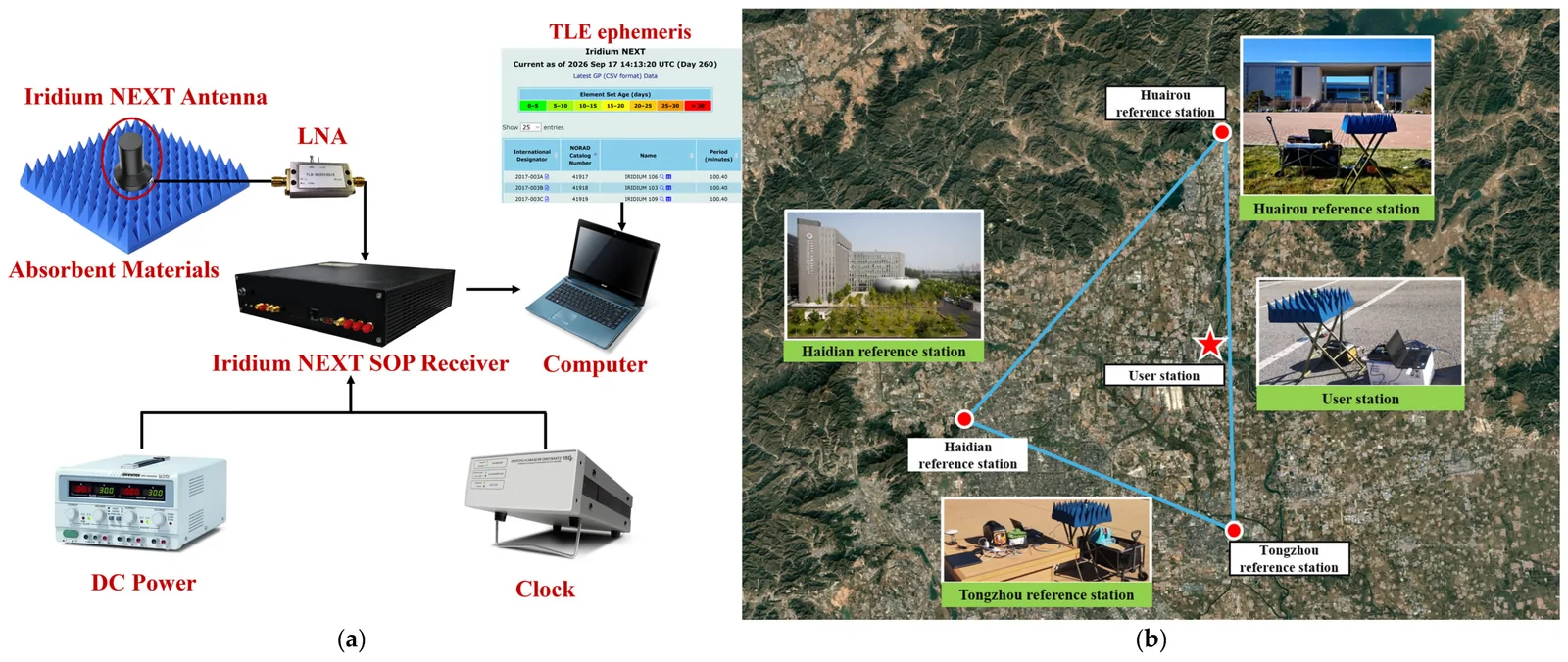
Experimental setup: (**a**) Iridium NEXT Signal Acquisition and Processing Platform. (**b**) Distribution of Ground Reference Stations and the User Station.

The Iridium NEXT signal-of-opportunity acquisition and processing platform is shown in Figure 4a. It mainly consists of an Iridium NEXT antenna, an Iridium NEXT signal receiver, a clock unit, a DC power supply, and a workstation (laptop). The receiver was developed in-house, and the time reference is provided by a GNSS timing receiver. The laptop is used for frequency estimation of the Iridium NEXT signals. RF-absorbing material is placed around the antenna to reduce multipath interference. A low-noise amplifier (LNA) is connected between the antenna and receiver to reduce noise effects. A Java program on the workstation performs Doppler-based LEO satellite orbit determination using TLE data as the initial orbit prior.

The station layout is shown in Figure 4b. Three reference stations and one user station were deployed. The Haidian, Huairou, and Tongzhou reference stations were located at (40.0709° N, 116.2743° E), (40.4072° N, 116.6746° E), and (39.9081° N, 116.6786° E), respectively. The user station was located at (40.1435° N, 116.6756° E).

To further characterize the current station-network geometry, the Haidian–Huairou, Haidian–Tongzhou, and Huairou–Tongzhou baselines are approximately 50.5, 39.0, and 55.4 km, with corresponding mean satellite LOS separation angles of 1.85°, 1.43°, and 2.01°, respectively. These results indicate measurable LOS diversity within the regional network, although the mean separation angles are relatively small. For the proposed adaptive weighting method, station-network geometry affects the Doppler measurement Jacobians and thus the relative geometric sensitivities of the observations; greater LOS diversity may provide more complementary directional constraints.

During the experiment, multiple Iridium NEXT satellites were observed, among which 42 satellites had repeated pass observations, yielding 92 pass observations. After excluding eight passes with insufficient observation duration, 84 effective passes were retained for subsequent processing. The average effective arc length was 11.43 min, and the Doppler sampling interval was 4.32 s. The time difference between each observation epoch and the corresponding TLE epoch had a mean value of 13.83 h. As a representative example of the jointly estimated clock-drift parameters, for the orbit-determination arc of satellite NORAD 43075, the estimated relative clock-drift parameters for the Haidian, Huairou, and Tongzhou reference stations were −5.10 × 10^−10^, −6.46 × 10^−10^, and −5.22 × 10^−10^, respectively, corresponding to equivalent frequency biases of approximately −0.83, −1.05, and −0.85 Hz at the Iridium NEXT carrier frequency. These estimates illustrate the magnitude of the clock-related frequency terms jointly estimated with the orbital state in the multi-station solution. The main algorithm parameters used in the experiments are listed in Table 1.

Among the parameters in Table 1, the adaptive adjustment-factor bounds and the regularization coefficient directly control the weight-adjustment behavior. The lower and upper bounds prevent excessive amplification or suppression of individual measurement weights relative to the a priori weights, while the regularization coefficient controls the trade-off between reducing the velocity-projection uncertainty metric and preserving the a priori weighting structure. The values were empirically selected through preliminary experimental tuning and were then kept fixed for all comparative experiments.

### 5.2. Iterative Behavior of Diagonally Scaled LM

**Figure 5:**
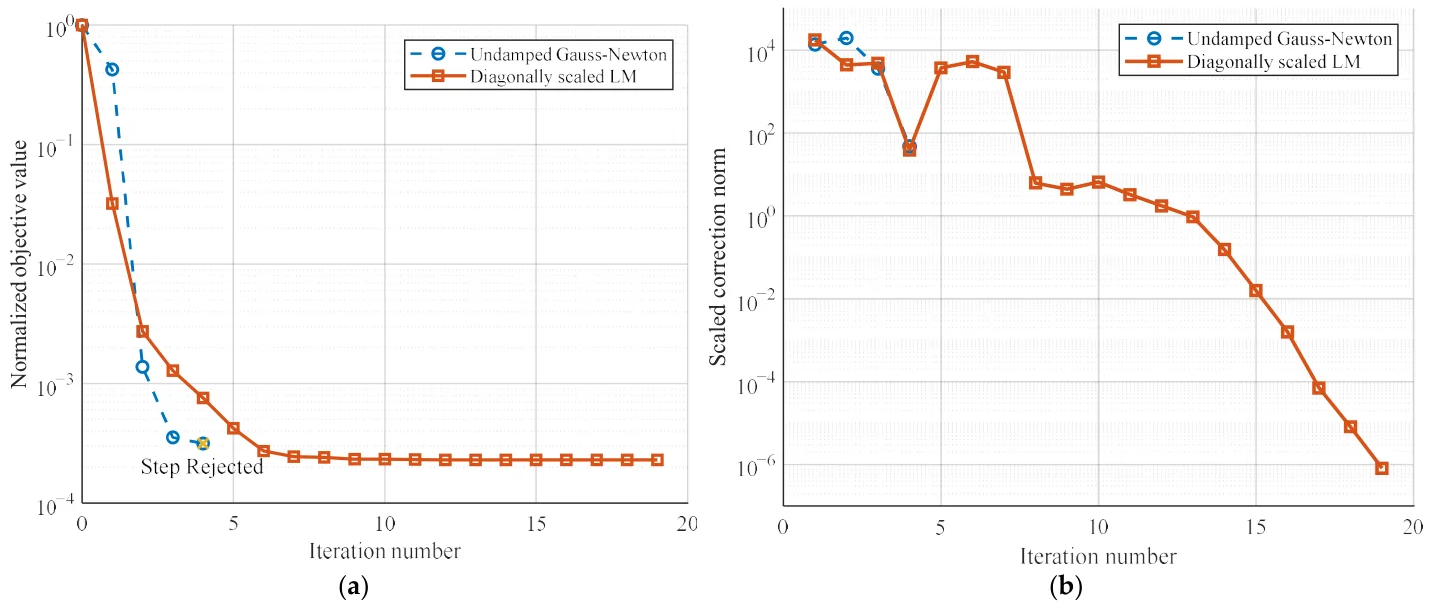
Effect of Diagonally Scaled LM on Orbit Determination Iterations: (**a**) Normalized Objective Value. (**b**) Scaled Correction Norm.

To examine the effect of diagonally scaled LM on the orbit determination state-update process, a measured multi-station Doppler arc from the Iridium NEXT satellite with North American Aerospace Defense Command (NORAD) catalog number 43075 was selected. Orbit determination was performed using undamped Gauss–Newton and diagonally scaled LM state updates under the same initial orbital state, orbital dynamics model, measurement data, measurement weights, and convergence criterion. Both methods used fixed equal measurement weights to exclude the effect of adaptive weighting on the iterative behavior. The normalized objective value is defined as the ratio of the current objective value to its initial value. The scaled-correction norm is calculated using the 2-norm. The results are shown in Figure 5.

Figure 5a shows that the undamped Gauss–Newton method reduced the normalized objective value during the first four iterations. However, the iteration terminated when the subsequent trial update failed to produce a further reduction. In contrast, diagonally scaled LM continued to reduce and stabilize the objective through adaptive damping. As shown in Figure 5b, the scaled correction norm had not converged when the undamped method terminated. With LM, the norm briefly increased but subsequently decreased to the convergence threshold. These results indicate that diagonally scaled LM can provide acceptable state corrections when undamped updates fail, thereby improving the stability of orbit determination iterations.

Accordingly, all three methods use the same diagonally scaled LM state update. Equal, a priori, and adaptive weighting are then evaluated through the residual and positioning analyses presented in the following subsections.

### 5.3. Multi-Station Doppler Measurements

On 16 December 2024, downlink signals from multiple Iridium NEXT satellites were collected at three reference stations. Doppler measurements were then obtained at each epoch through frequency estimation [51]. Satellites with NORAD catalog numbers 43070 and 43079 were selected to illustrate the characteristics of the measured multi-station Doppler data.

**Figure 6:**
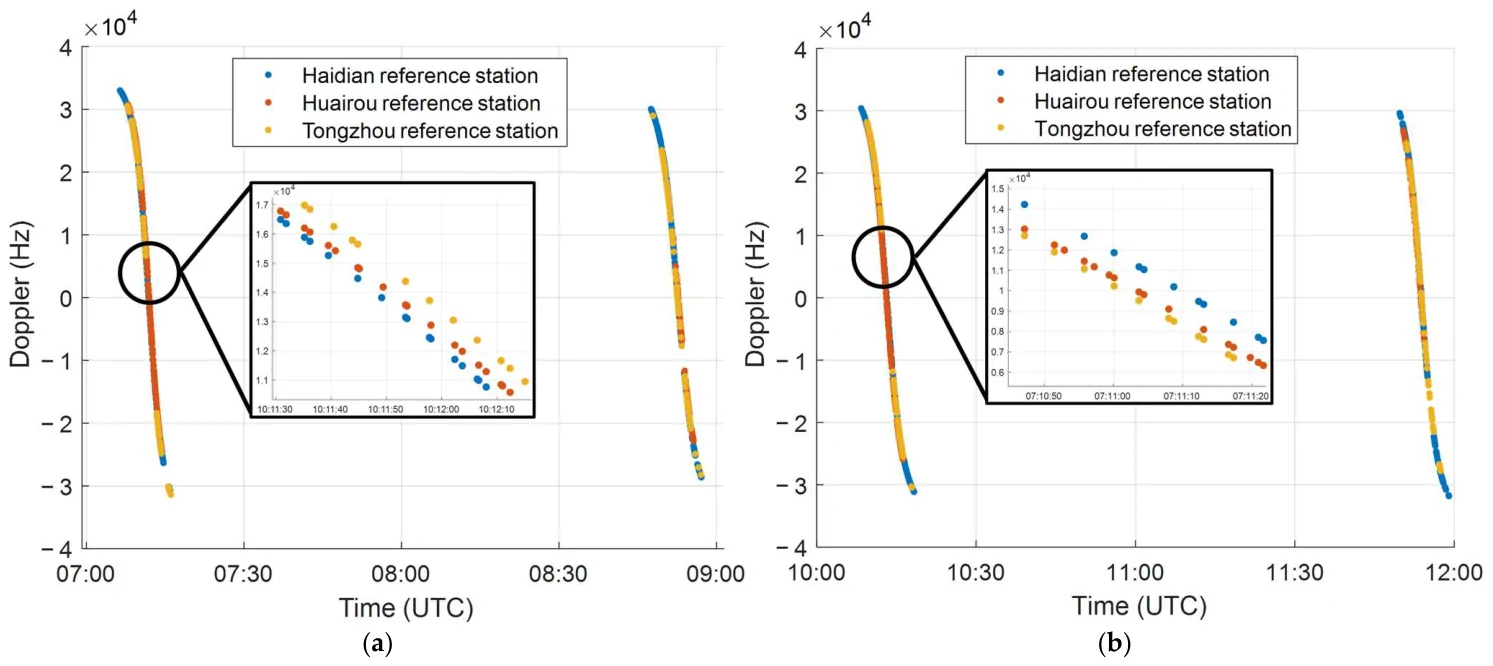
Doppler Measurements from Iridium NEXT Satellites: (**a**) Doppler Measurements for NORAD 43070. (**b**) Doppler Measurements for NORAD 43079.

Figure 6 shows Doppler measurements of Iridium NEXT satellites NORAD 43070 and 43079 at the Haidian, Huairou, and Tongzhou stations. During each pass, the Doppler shift varies continuously as the LOS range rate changes. Measurements from different stations follow similar trends but differ in magnitude, slope, and effective observation interval because of their different LOS geometries.

### 5.4. Analysis of Adaptive Weight Allocation Characteristics

To analyze the weight-allocation characteristics of the proposed method, a representative measured pass was selected. For the Haidian station, the elevation-based a priori weight, adaptive adjustment factor, and final measurement weight are presented. The relationship between the sensitivity of the velocity-projection uncertainty metric and the adaptive adjustment factor is also presented for the three reference stations.

**Figure 7:**
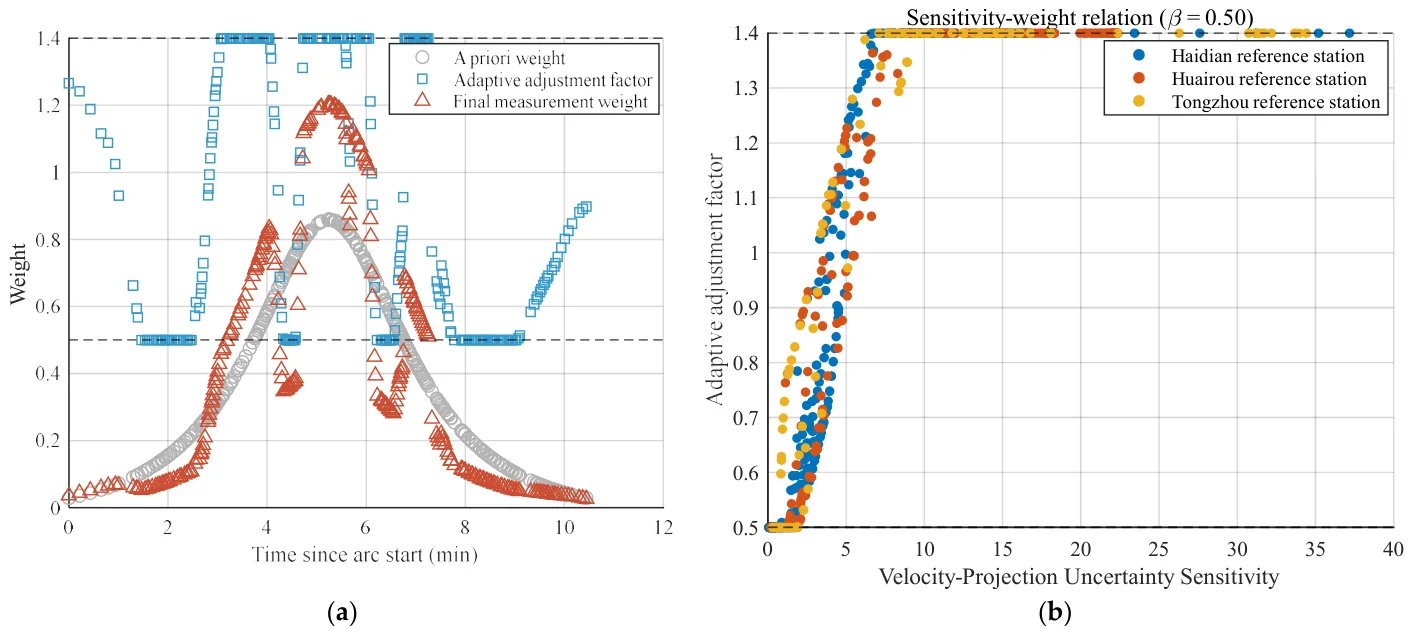
Adaptive Weight Allocation Characteristics during a Representative Measured Pass: (**a**) Weight Components at the Haidian Station. (**b**) Relationship between Sensitivity and Adaptive Adjustment Factor.

Figure 7a shows that the elevation-based a priori weight varies smoothly with the satellite elevation angle, whereas the adaptive adjustment factor exhibits multiple peaks during the pass. This variation is mainly associated with the time-varying observation geometry during the pass. The final measurement weight is the product of the elevation-based a priori weight and the adaptive adjustment factor. It therefore incorporates both measurement quality and geometric contribution. Figure 7b shows an overall positive relationship between the velocity-projection uncertainty sensitivity and the adaptive adjustment factor. Measurements with lower sensitivities generally receive smaller adjustment factors, whereas those with higher sensitivities tend to receive larger factors. Of all adjustment factors, 22.7% reach the lower bound, 37.5% reach the upper bound, and the remaining 39.8% lie between the two bounds. The measurements reaching the lower and upper adjustment-factor bounds have mean elevation angles of 31.7° and 48.4°, respectively, showing that the upper-bound group tends to have higher elevations on average. However, elevation alone does not determine the adaptive adjustment. The median velocity-projection sensitivity of the upper-bound group is approximately 18.2 times that of the lower-bound group, and the Pearson correlation coefficient between the sensitivity and the adjustment factor is 0.718. These results indicate that, in addition to the measurement-quality information represented by the elevation-based a priori weights, the adaptive adjustment further differentiates the measurements according to their geometric contribution to the Doppler-relevant directional constraint.

### 5.5. Effect of the Number of Reference Stations

To further examine the effect of the number of reference stations, single-station OD was first attempted using the measurements from each reference station separately under the same estimation settings. However, none of the single-station solutions satisfied the prescribed convergence criterion, and therefore unconverged solutions were not used for quantitative comparison. As a supplementary evaluation, three satellites were selected from the cases for which both the two-station and three-station OD solutions converged stably and sufficient valid observations were available for a consistent comparison. Equal weighting was adopted in both cases, while the initial orbit, dynamical model, state-update strategy, and convergence criterion were kept unchanged. For the three selected satellites, the Haidian–Huairou, Haidian–Tongzhou, and Huairou–Tongzhou station pairs were used sequentially for the two-station solutions, respectively. The corresponding positioning errors are summarized in Table 2.

For the selected cases, the three-station solutions yielded lower 2D positioning errors than the corresponding two-station solutions, with reductions ranging from 21.95% to 36.23%. These results suggest that, in the examined examples, incorporating observations from a third reference station can provide additional geometric constraints and improve the utility of the estimated orbital states for subsequent positioning.

### 5.6. In-Arc Post-Fit Doppler Residual Analysis

To further assess the in-arc fitting consistency of the three OD solutions, the theoretical Doppler values were recomputed using the converged orbital states and station clock-drift parameters for the multi-station measurements used in orbit determination. The corresponding unweighted post-fit residuals were then obtained, and their mean, RMS, and STD are summarized in Table 3.

The equally weighted solution yields the smallest unweighted RMS, which is consistent with its direct minimization of the unweighted residual sum of squares. After introducing the elevation-based a priori weights, the RMS increases slightly because the relative contributions of individual observations are redistributed during estimation. The subsequent adaptive adjustment reduces the RMS relative to the a priori weighted solution, bringing it close to that of the equally weighted solution. Meanwhile, the nearly identical STD values indicate that the overall residual dispersion remains essentially unchanged. These results show that the three methods achieve comparable in-arc fitting performance, while the adaptively weighted solution maintains a fitting level close to that of the equally weighted solution. The benefit of adaptive weighting is more clearly reflected in the subsequent-pass user-station Doppler residuals and positioning results.

### 5.7. User-Station Positioning Validation and Analysis

To evaluate the positioning utility of the adaptively weighted orbit determination (OD) solution, comparative user-station positioning experiments were conducted using height aiding from the barometric sensor integrated into the user equipment. Multi-station Doppler measurements from the first effective pass were used for orbit determination, and the estimated orbital states were propagated to the subsequent effective pass for positioning validation, with an average propagation interval of 1.58 h between the orbit-determination pass and the subsequent positioning-validation pass. For each of the 500 positioning trials, three satellites were randomly selected, and a continuous 600-s observation interval was extracted from the effective arc of each satellite using a randomly selected starting epoch. For the static user station, the velocity was set to zero, and the user position and a common receiver clock-drift parameter were jointly estimated.

**Figure 8:**
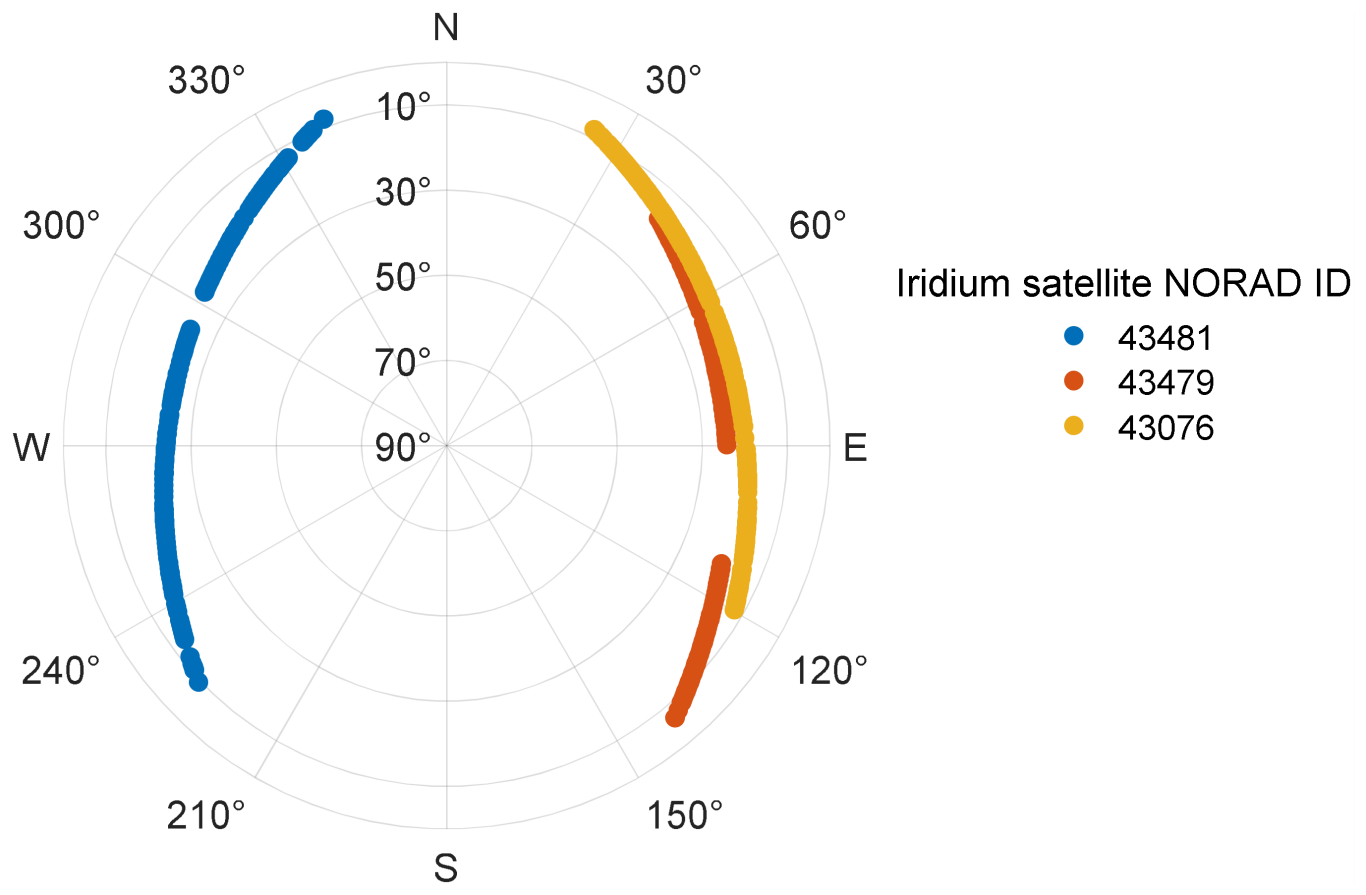
Sky Plot for a 10-min Positioning Trial.

Figure 8 shows the sky plot of the Iridium NEXT satellites observed at the user station during a 10-min positioning trial. The satellites are spatially distributed across the sky, providing geometric constraints for horizontal position estimation at the user station.

The same satellite combination and observation intervals were used for all orbit solutions within each trial. The mean observation-data overlap ratio among the 500 trials was 33.8%. Each trial was processed using the TLE-derived orbit and the equally weighted, a priori weighted, and adaptively weighted OD solutions. The three OD methods differed only in their weighting strategies and otherwise used the same LM mechanism, parameter settings, initial orbital state, orbital dynamics model, measurement data, and convergence criterion. The east, north, and 2D positioning RMSEs were then computed over the 500 trials.

**Figure 9:**
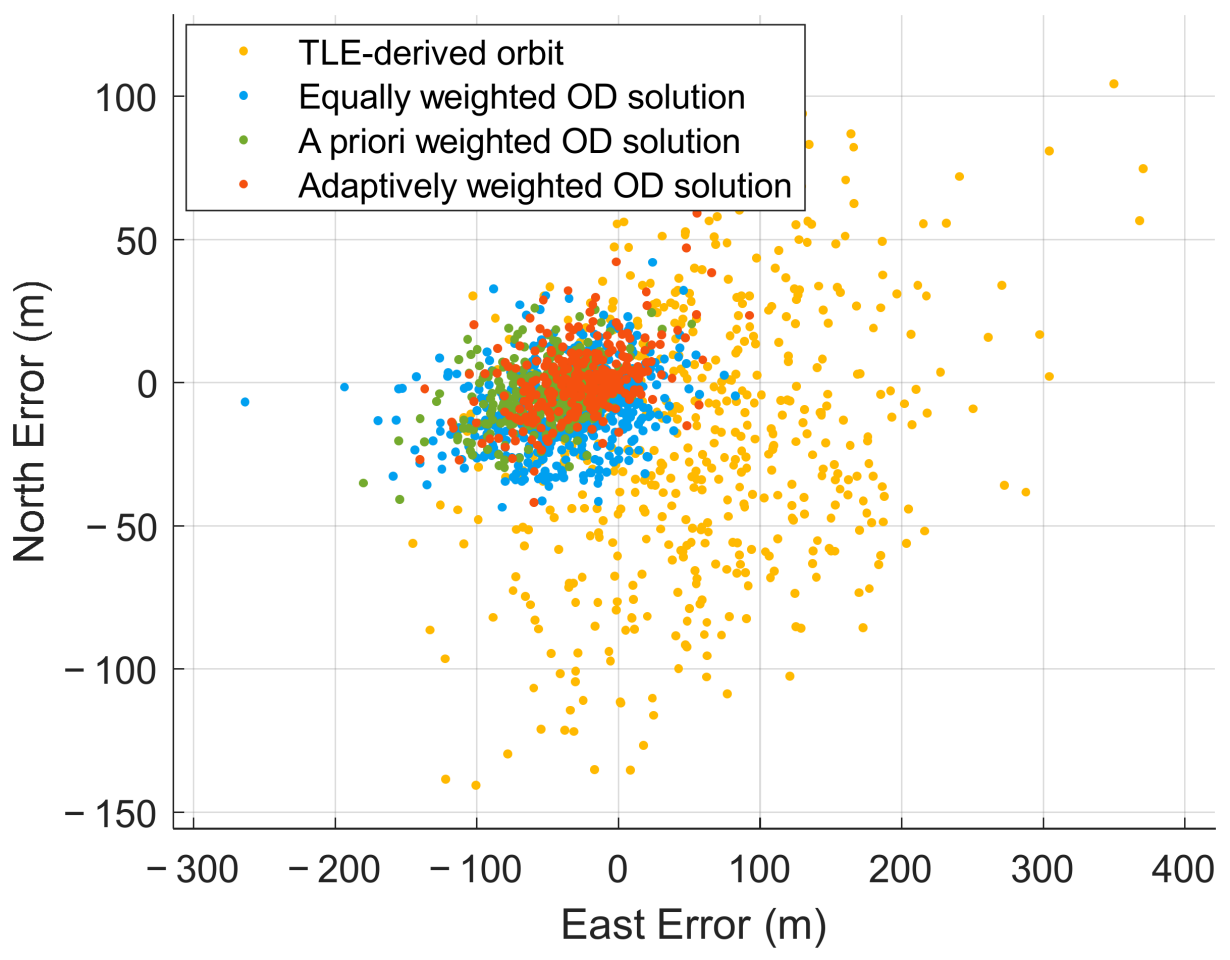
Distribution of 2D Positioning Errors.

Figure 9 shows the 2D positioning errors for 500 10-min observation trials. In this experiment, errors obtained with the TLE-derived orbit were widely dispersed and shifted eastward. The equally weighted OD solution reduced both the eastward offset and overall dispersion. The distribution contracted further with the a priori weighted OD solution and became more concentrated near the origin with the adaptively weighted OD solution. These results indicate that OD solutions obtained from multi-station Doppler measurements improved user-station positioning. Introducing adaptive weight adjustment on top of a priori weighting further reduced the user-station positioning errors.

Table 4 summarizes the user-station 2D positioning errors for 500 10-min observation trials using four sets of orbital states. In this experiment, the TLE-derived orbit produced a 2D RMSE of 115.51 m. The equally weighted OD solution reduced it to 54.80 m, a reduction of 52.56% relative to the TLE-derived orbit. The a priori and adaptively weighted OD solutions further reduced the 2D RMSE to 49.20 m and 41.30 m, representing reductions of 10.22% and 16.06% relative to the equally weighted and a priori weighted solutions, respectively. The adaptively weighted OD solution achieved an overall reduction of 64.25% relative to the TLE-derived orbit. These results indicate that multi-station Doppler OD improved user-station positioning. Introducing adaptive weight adjustment on top of a priori weighting further reduced the positioning errors.

### 5.8. User-Station Doppler Residual Analysis

**Figure 10:**
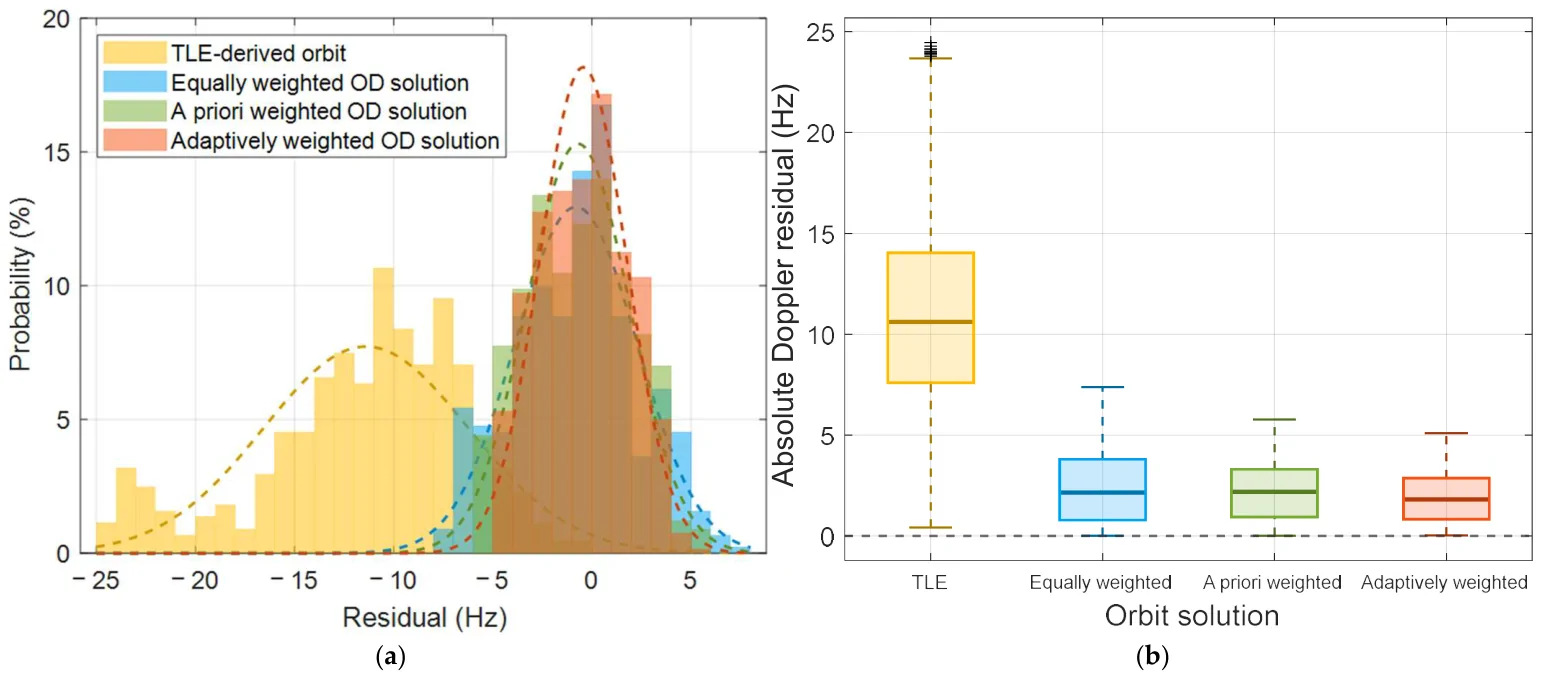
User-Station Doppler Residuals for Four Orbit Solutions over 500 10-min trials: (**a**) Histogram of Doppler Residuals. (**b**) Box Plot of Absolute Doppler Residuals.

To provide an observation-domain assessment independent of the measurements used for orbit determination, Doppler residuals were evaluated at the user station during the subsequent pass. These user-station measurements were not involved in the orbit-determination process. Residuals between the theoretical and measured Doppler values were then computed for the four orbit solutions, and their mean, root mean square (RMS), and standard deviation (STD) were calculated. Figure 10 shows the residual distributions over 500 10-min observation trials. Figure 10a presents the residual histograms, Figure 10b presents box plots of the absolute residuals, and the corresponding statistics are listed in Table 5.

Figure 10a shows that the residuals obtained with the TLE-derived orbit were mainly negative and clearly offset from zero. The equally weighted OD solution shifted the residuals toward zero, while the a priori and adaptively weighted OD solutions progressively reduced their dispersion. Figure 10b shows that the adaptively weighted OD solution produced smaller absolute residuals and a narrower interquartile range than the equally weighted OD solution. Table 5 shows that the residual mean progressively approached zero. The adaptively weighted OD solution yielded the mean closest to zero, indicating a further reduction in the overall residual offset. The residual RMS decreased from 3.19 Hz for the equally weighted OD solution to 2.70 Hz for the a priori weighted OD solution and further to 2.27 Hz for the adaptively weighted OD solution. Compared with the a priori and equally weighted OD solutions, the adaptively weighted OD solution reduced the RMS by 15.93% and 28.84%, respectively. The residual STD also decreased progressively. Together, the reductions in RMS and STD indicate decreases in both residual magnitude and dispersion. These results indicate closer agreement between the theoretical and measured Doppler values for the adaptively weighted OD solution.

The positioning results and Doppler residual analysis indicate the application utility of the estimated orbital states in terms of positioning errors and agreement with the Doppler measurements.

## 6. Conclusions

An adaptively weighted multi-station Doppler orbit determination method is proposed to mitigate the effect of TLE-derived orbital-state errors on Doppler positioning with Iridium NEXT signals of opportunity. The method adaptively weights multi-station, multi-epoch Doppler measurements according to measurement quality and geometric constraint strength, while an LM-type damping mechanism with diagonal scaling stabilizes the BLS state update. Experimental results using real measurements showed that the estimated orbital states yielded a user-station 2D RMSE of 41.30 m. This value was 16.06% lower than that obtained with the a priori weighted OD solution, 24.64% lower than that obtained with the equally weighted OD solution, and 64.25% lower than that obtained with the TLE-derived orbit. These results indicate that adaptive weighting further reduced user-station positioning errors. The current experiments were limited to a station network in a single region and consecutive Iridium NEXT passes. The estimated orbital states were evaluated indirectly using in-arc post-fit Doppler residuals, subsequent-pass user-station Doppler residuals, and positioning errors. Absolute orbit determination accuracy has not yet been validated against an independent precise reference orbit. The weight optimization objective focuses on the uncertainty of the satellite velocity projected along the LOS from the station network centroid to the satellite. Its performance may depend on the station geometry and the user’s location relative to the network. The current weight model also does not explicitly account for outliers or the time-varying characteristics of atmospheric, timing, and frequency errors. Future work will use measurements from multiple passes and regions to investigate arc-to-arc state continuity constraints, robust weighting, and long-term orbit prediction. The applicability of the proposed method under different observation conditions will also be evaluated.

## Figures and Tables

**Table 1:** Algorithm Parameters.

Parameter	Symbol	Value
Adaptive Adjustment Factor Range	$\left[w_{\min},w_{\max}\right]$	[0.5, 1.4]
Regularization Coefficient	β	0.5
Initial Line-Search Step	$\alpha_{0}$	1.0
Adjustment-Factor Convergence Tolerance	$\eta_{w}$	1.0 × 10^−6^
Initial LM Damping	$\mu^{\left(0\right)}$	1.0 × 10^−2^
LM Adjustment Factor	γ	10
Scaled State-Correction Tolerance	$\varepsilon_{x}$	1.0 × 10^−6^

**Table 2:** Comparison of 2D Positioning Errors Using Two-Station and Three-Station OD Solutions.

NORAD ID	Two-Station Pair	Two-Station Error (m)	Three-Station Error (m)	Error Reduction (%)
43075	Haidian–Huairou	340.15	216.90	36.23
43070	Haidian–Tongzhou	280.97	213.12	24.15
43481	Huairou–Tongzhou	158.17	123.45	21.95

**Table 3:** Unweighted In-Arc Post-Fit Doppler Residual Statistics for Three OD Solutions.

Orbit Solution	Mean (Hz)	RMS (Hz)	STD (Hz)
Equally weighted	−0.283	1.615	1.590
A priori weighted	−0.360	1.629	1.589
Adaptively weighted	−0.305	1.618	1.589

**Table 4:** User-Station Positioning Results Using Four Orbit Solutions.

Orbit Solution	East RMSE (m)	North RMSE (m)	2D RMSE (m)	2D RMSE Reduction (%)
TLE-derived	101.57	54.99	115.51	0 (baseline)
Equally weighted	51.98	17.34	54.80	52.56
A priori weighted	47.38	13.25	49.20	57.41
Adaptively weighted	39.62	11.65	41.30	64.25

**Table 5:** User-Station Doppler Residual Statistics for Four Orbit Solutions.

Orbit Solution	Mean (Hz)	RMS (Hz)	STD (Hz)
TLE-derived	−11.40	12.51	5.16
Equally weighted	−0.75	3.19	3.10
A priori weighted	−0.65	2.70	2.62
Adaptively weighted	−0.37	2.27	2.24

## Data Availability

All experimental data were collected using our equipment and are available upon request by contacting M.L.

## Abbreviations

The following abbreviations are used in this manuscript:

LEO	Low Earth Orbit
TLE	Two-Line Element
BLS	Batch Least Squares
LOS	Line of Sight
LM	Levenberg–Marquardt
2D	Two-Dimensional
RMSE	Root Mean Square Error
GNSS	Global Navigation Satellite System
SOP	Signals of Opportunity
SGP4	Simplified General Perturbations 4
EME2000	Earth Mean Equator and Equinox of J2000.0
STM	State Transition Matrix
ITRF	International Terrestrial Reference Frame
IERS	International Earth Rotation and Reference Systems Service
LNA	Low-Noise Amplifier
NORAD	North American Aerospace Defense Command
OD	Orbit Determination
RMS	Root Mean Square
STD	Standard Deviation
